# The Impact of Parental Preconception Nutrition, Body Weight, and Exercise Habits on Offspring Health Outcomes: A Narrative Review

**DOI:** 10.3390/nu16244276

**Published:** 2024-12-11

**Authors:** Alireza Jahan-Mihan, Jamisha Leftwich, Kristin Berg, Corinne Labyak, Reniel R. Nodarse, Sarah Allen, Jennifer Griggs

**Affiliations:** 1 Department of Nutrition and Dietetics, University of North Florida, 1 UNF Dr., Jacksonville, FL 32224, USA; j.leftwich@unf.edu (J.L.); k.berg@unf.edu (K.B.); c.labyak@unf.edu (C.L.); n01569362@unf.edu (R.R.N.); 2 Greenleaf Behavioral Health, 2209 Pineview Dr., Valdosta, GA 31602, USA; sarahallenrdn@gmail.com; 3 AdventHealth Ocala, 1500 SW 1st Ave, Ocala, FL 34471, USA; jennifer.griggs@adventhealth.com

**Keywords:** paternal preconception, maternal preconception, offspring’s health, diet, obesity

## Abstract

An increasing number of studies highlight the critical role of both maternal and paternal nutrition and body weight before conception in shaping offspring health. Traditionally, research has focused on maternal factors, particularly in utero exposures, as key determinants of chronic disease development. However, emerging evidence underscores the significant influence of paternal preconception health on offspring metabolic outcomes. While maternal health remains vital, with preconception nutrition playing a pivotal role in fetal development, paternal obesity and poor nutrition are linked to increased risks of metabolic disorders, including type 2 diabetes and cardiovascular disease in children. This narrative review aims to synthesize recent findings on the effects of both maternal and paternal preconception health, emphasizing the need for integrated early interventions. The literature search utilized PubMed, UNF One Search, and Google Scholar, focusing on RCTs; cohort, retrospective, and animal studies; and systematic reviews, excluding non-English and non-peer-reviewed articles. The findings of this review indicate that paternal effects are mediated by epigenetic changes in sperm, such as DNA methylation and non-coding RNA, which influence gene expression in offspring. Nutrient imbalances during preconception in both parents can lead to low birth weight and increased metabolic disease risk, while deficiencies in folic acid, iron, iodine, and vitamin D are linked to developmental disorders. Additionally, maternal obesity elevates the risk of chronic diseases in children. Future research should prioritize human studies to explore the influence of parental nutrition, body weight, and lifestyle on offspring health, ensuring findings are applicable across diverse populations. By addressing both maternal and paternal factors, healthcare providers can better reduce the prevalence of metabolic syndrome and its associated risks in future generations.

## 1. Introduction

For decades, maternal health has been the primary focus of studies examining the origins of chronic diseases and poor offspring health outcomes, with in utero exposures considered the dominant determinant [1]. However, emerging research suggests that both maternal and paternal nutrition and body weight before conception can significantly influence the long-term health outcomes of offspring [2,3]. Preconception nutrition, body weight, and lifestyle choices of both parents are increasingly recognized as critical in shaping long-term health trajectories.

The impact of maternal preconception health, particularly nutrition and body weight, on fetal development and the offspring’s predisposition to metabolic abnormalities has been widely studied [2,4]. Meanwhile, recent evidence reveals the vital contribution of paternal factors, such as body composition and dietary habits, to offspring metabolic health. Paternal obesity and poor nutrition have been associated with alterations in sperm quality and epigenetic markers, leading to an elevated likelihood of metabolic disorders, including type 2 diabetes and cardiovascular diseases, in offspring [2,3]. Inversely, paternal exercise has shown protective effects, improving offspring’s metabolic health. Parental physical activity, including exercise by the father, has been demonstrated to positively influence tissue metabolism and serum metabolite profiles in offspring, helping regulate glucose tolerance and fat metabolism, which are key markers for metabolic health [5].

Paternal health, including preconception nutrition, weight, and metabolic status, profoundly influences offspring outcomes [6]. Paternal programming suggests that a father’s preconception environment impacts seminal plasma and sperm cells, influencing embryonic development and the long-term metabolic and reproductive health of offspring [4]. Numerous studies, primarily in rodent models, have linked paternal diet, obesity, and low sperm quality to an increased risk of cardiometabolic disorders, such as obesity and type 2 diabetes, in offspring [5,7,8,9]. Bromfield et al. [10] found that the absence of seminal plasma decreased the expression of embryotrophic factors, including Leukemia Inhibitory Factor (LIF), Colony-Stimulating Factor 2 (CSF2), Interleukin 6 (IL6), and Epidermal Growth Factor (EGF). Simultaneously, levels of apoptosis-inducing factors like TRAIL increased in the oviduct, potentially impacting preimplantation embryonic development. They also found that embryos formed in female reproductive tracts that lacked exposure to seminal plasma displayed abnormalities from the early cleavage stages; however, these issues were partially mitigated through in vitro culture. The authors suggested the significant role of paternal seminal fluid composition in influencing fetal development and the health of male offspring [11]. Sperm can carry chemical modifications (like DNA methylation, histone modifications, and small non-coding RNAs) that influence gene expression in the offspring without altering the DNA sequence. These changes can be triggered by the father’s environment, such as diet, stress, and exposure to toxins [12]. During early embryonic development, most epigenetic marks are reset, but some modifications from the father’s sperm can “escape” this process, potentially affecting the child’s development and health throughout their life [13]. Specifically, paternal obesity and diabetes have been linked to altered sperm DNA methylation and changes in small non-coding RNAs, leading to adverse effects on offspring metabolism, growth, and susceptibility to conditions such as obesity and diabetes [14]. Notably, these epigenetic modifications can be passed across multiple generations, amplifying the long-term impact on population health [15]. Recent studies also suggest that parental undernutrition or overnutrition prior to conception can result in lasting changes in gene expression in offspring, leading to increased risk for cardiovascular disease, impaired glucose regulation, and other non-communicable diseases later in life [14]. Furthermore, some studies have begun exploring the role of maternal and paternal micronutrient intake, such as vitamin D, zinc, and folate, on reproductive success and offspring health outcomes, highlighting the potential for targeted nutritional interventions [16].

Understanding the interplay between maternal and paternal health is essential for developing early interventions that address the root causes of intergenerational health disparities. Therefore, the goal of this review is to examine how the preconception health and lifestyle choices of both parents impact offspring outcomes, emphasizing the need for comprehensive strategies to reduce the burden of metabolic disorders in future generations.

## 2. Methodology

This narrative review was conducted in the following steps: search execution, evaluation of abstracts and full-texts, and synthesis of findings. Searches were performed using PubMed, UNF One Search (via the UNF Library), ScienceDirect, and Google Scholar to identify studies relevant to the review’s objectives. The final search, completed in November 2024, included international articles and online reports published in English. Eligible study designs included Randomized Controlled Trials (RCTs), prospective and retrospective studies, animal studies, cohort studies, and systematic reviews. Keywords such as “paternal preconception”, “maternal preconception”, “parental preconception”, “offspring health”, “diabetes”, “dyslipidemia”, “obesity”, “metabolic syndrome”, “epigenetics”, and “immune system” were used to retrieve relevant literature. After conducting the search, abstracts were screened to confirm relevance to the review topic. Duplicates were removed, and the remaining abstracts were further assessed for adherence to inclusion criteria. Studies were excluded if they were non-English publications or lacked peer review.

## 3. The Impact of Parental Health During Preconception on Offspring

### 3.1. The Impact of Parental Diet During Preconception

Parental diet during preconception plays a vital role in shaping the health trajectory of offspring. The nutritional status and dietary choices of both parents during the preconception period can impact fetal development, gene expression, and health outcomes in later life in offspring. Additionally, the preconception diet can induce maternal and paternal epigenetic changes, potentially influencing gene expression patterns that impact growth, immune function, and disease susceptibility throughout the child’s life [17].

#### 3.1.1. The Impact of Maternal Diet

Growing research highlights the significance of maternal nutrition during the preconception period in the developmental outcomes of offspring and their subsequent health. The preconception period, defined as the months leading up to conception, is increasingly recognized as a crucial window during which maternal nutritional status may influence the outcome of pregnancy and the health of the offspring in later life. Maternal nutritional reserves established before conception play a crucial role in supporting early embryonic development, the function of the placenta, and fetal growth. Nutritional shortage or imbalances during this timeframe may negatively impact the birth outcome, such as with low birth weight and Small-for-Gestational-Age (SGA) infants, which may result in increased risks of cardiovascular disease, and diabetes later in life [18].

Micronutrient deficiencies during the preconception period, including folic acid, iron, iodine, and vitamin D, can be very consequential during development and shape the health of offspring [19]. Folic acid, in particular, is critical for preventing neural tube defects and promoting healthy brain development [20]. Micronutrient deficiencies during the preconception period, such as low levels of iron, iodine, folic acid, and vitamin D, can have serious effects on fetal development and influence the long-term health of the offspring [19]. Folic acid is essential, as it helps prevent neural tube defects like spina bifida and supports healthy brain development and DNA repair and DNA synthesis [20,21].

The role of parental folate deficiency in the preconception period is also investigated. Folate is involved in the synthesis of S-Adenosylmethionine (SAM), which is crucial for several biological procedures, including protein and lipid metabolism, the methylation of DNA and RNA, and the synthesis of hormones and nucleotides [22]. Maternal folate deficiency is linked to Neural Tube Defects (NTDs) and other developmental changes in offspring [23]. Reduced maternal folate levels are also associated with issues including low birth weight, intrauterine growth restriction, and altered glucose metabolism, as indicated by hyperglycemia and insulin resistance in offspring [24]. Emerging evidence suggests that paternal folate intake can also prompt the risk of diseases in offspring. A recent study found that the offspring of male rats that were folate-deficient were more prone to developing anxiety and depression-like traits [25]. In a study by Lambrot et al. in 2013, low paternal folate intake during the periconceptional period altered sperm epigenetics and led to lower pregnancy rates, abnormal placental development, and post-implantation loss [26]. Folate levels also affect human reproductive health, particularly gestation duration, with a recommended folate intake of 4 mg/day for men [27]. In mice, folate deficiency in fathers is associated with a higher incidence of developmental irregularities, such as limb defects, delays in muscle and skeletal development, and craniofacial malformations. Additionally, the offspring of folate-deficient fathers exhibit delayed meiosis by postnatal day 12, although no significant changes in sperm count, spermatogenesis, or testis morphology in adulthood were observed [26]. However, other studies have shown impaired spermatogenesis and reduced sperm counts in similar conditions [28,29]. Paternal folate deficiency has also been associated with altered placental folate transport, DNA methylation, and the expression of Igf-2 in the fetal brain [30,31,32].

Iron deficiency, on the other hand, may reduce oxygen supply to developing tissues, leading to risks like premature birth, low birth weight, and cognitive issues in children [33]. Iodine is crucial for thyroid function, which plays an important role in the development of the brain; low iodine levels have been associated with cognitive delays and developmental issues [34]. Additionally, insufficient vitamin D during preconception or pregnancy has been linked to risks including gestational diabetes, preeclampsia, and poor bone development in the baby [35]. Therefore, ensuring proper intake of these essential nutrients before conception is key to supporting maternal health as well as the optimal fetal development [36].

Adequate and balanced protein and calorie intake during preconception are vital for building nutritional reserves that support early fetal development. A low-protein or low-calorie diet can hinder placental development and nutrient transport to the fetus, leading to growth restrictions [37]. Expanding on the role of adequate and balanced protein and calorie intake during the preconception period, research suggests that beyond supporting placental development, these macronutrients are crucial for ensuring proper cell division and organogenesis in the early stages of development of fetus. Insufficient protein intake can impair the synthesis of amino acids necessary for tissue growth, while inadequate calorie consumption may limit the energy available for fetal metabolic demands, leading to potential health complications in long-term for the child, including metabolic disorders and impaired cognitive development [38]. Furthermore, balanced nutrition is key to promoting maternal health, as it helps in maintaining optimal body weight and reduces the risk of complications including preeclampsia or gestational diabetes, which can also adversely affect fetal growth [36]. Not only does a protein and calorie-deficient diet hinder placental function, but it also compromises the mother’s ability to support the increasing energy demands during pregnancy. This can result in Intrauterine Growth Restriction (IUGR), predisposing the offspring to chronic diseases like hypertension and cardiovascular diseases in adulthood [39]. Recent studies further emphasize the need for dietary diversity during the preconception phase, noting that the eating of a variety of healthy, nutrient-dense foods can enhance reproductive health, improve fertility outcomes, and prepare the body to fulfill the dietary needs during pregnancy [40,41].

A vegetarian diet during preconception can impact offspring health, offering benefits like antioxidants and phytochemicals that support fetal development and reduce chronic disease risks. However, it may lack critical nutrients such as vitamin B12, iron, zinc, iodine, and omega-3 fatty acids, essential for neurodevelopment, immune function, and growth. Deficiencies can lead to low birth weight, cognitive issues, and developmental delays. Supplementation or fortified foods and professional guidance are vital to ensure balanced nutrition and optimize outcomes [42].

A mother’s nutritional status before conception strongly correlated with birth weight, gestational age, and infant size [43]. Low body weight and BMI or malnutrition prior to pregnancy may lead to preterm delivery or SGA infants, both of which are linked to increased risks of infant death, developmental challenges, and long-term health issues [44]. Moreover, maternal preconception nutrition may impact offspring growth well beyond birth [45]. Children of malnourished mothers face a heightened risk of stunting, a condition that hinders growth and development that affects physical health, cognitive ability, and productivity throughout life [46]. Expanding further on the impact of maternal preconception nutritional status, it has become evident that a mother’s body composition and nutrient stores before pregnancy impact fetal growth and have enduring effects on the child’s metabolic health. Maternal malnutrition, including a lack of key micronutrients such as iron, folate, and vitamin D, is associated with Low Birth Weight (LBW) and Small-for-Gestational-Age (SGA) infants [47,48]. Poor preconception nutrition raises the likelihood of preterm birth, which poses significant challenges for neonatal health, such as respiratory distress and higher susceptibility to infections [49]. Additionally, a mother’s Body Mass Index (BMI) and nutritional status can “program” the health of her offspring, leading to long-term outcomes such as obesity, type 2 diabetes, and cardiovascular diseases in adulthood [50]. Stunting in children born to malnourished mothers, as previously noted, can lead to poor cognitive performance and lower educational attainment, thus affecting productivity and socioeconomic outcomes in adulthood [51]. Addressing preconception malnutrition is essential for breaking the cycle of poverty and poor health across generations [52].

Inadequate maternal nutrition before conception can elevate the risk of chronic conditions such as obesity, cardiovascular diseases, and type 2 diabetes in offspring later in life [53] via fetal programming. This process occurs when the mother’s nutritional status influences gene expression in the fetus, setting the stage for long-term health outcomes [54]. For instance, a mother’s preconception nutrient deficiencies or excesses can alter fetal metabolism and fat distribution, which may lead to metabolic disorders in adulthood [54]. Additionally, maternal undernutrition during the periconceptional period has been associated with insulin resistance and impaired glucose tolerance in offspring, raising the risk of metabolic syndrome [55]. Fathers also play a significant role in this process, as paternal obesity and poor dietary habits can alter sperm DNA integrity, potentially increasing the offspring’s vulnerability to obesity and metabolic syndrome [56]. Furthermore, lifestyle factors such as physical activity and stress levels before conception may compound these risks, with evidence suggesting that maternal stress can increase the likelihood of metabolic and cardiovascular diseases in offspring by affecting hormonal regulation and fetal development [57]. Therefore, improving parental nutrition and lifestyle before conception could be key to mitigating the risk of metabolic syndrome in future generations.

#### 3.1.2. The Impact of Paternal Diet

Paternal dietary choices and environmental exposures before conception can impact the health of future generations through various mechanisms, including epigenetics, such as DNA methylation, histone modification, and small non-coding RNAs (sncRNAs) [6].

Paternal High-Fat Diets (HFD) have been found to negatively impact the metabolic health of offspring. Animal studies demonstrate that consuming a high-fat diet before conception can lead to metabolic issues in offspring, including increased adiposity, insulin resistance, and impaired glucose metabolism [58]. It may also increase the risk of chronic diseases in offspring later in life. For instance, their offspring exhibited dysfunctional pancreatic β-cells and a higher risk of type 2 diabetes and obesity. Moreover, mice fathers with high-fat or nutrient-deficient diets exhibited changes in these reproductive components, leading to health issues in their offspring [6]. However, in another study, the offspring of high-fat diet-fed males exhibited better insulin sensitivity and improved insulin signaling pathways in skeletal muscle. These effects may reflect adaptive or compensatory responses to the paternal high-fat diet [59].

A paternal low-protein diet is also associated with various negative health outcomes in offspring, particularly affecting cardiovascular health and growth [60]. One study found that low-protein paternal diets result in higher risk of hypertension, impaired heart function, and altered lipid profiles in offspring, indicating a predisposition to cardiovascular diseases [61]. Notably, the effects were often sex-dependent, with male offspring exhibiting more pronounced metabolic and cardiovascular alterations than males [6]. In addition, poor folic acid intake in fathers during the preconception period may alter sperm DNA methylation, which has been associated with birth defects and compromised pregnancy outcomes [6]. Folic acid is vital for DNA methylation, an important epigenetic process that controls gene expression. A deficiency in folic acid can have significant effects on offspring health, especially during critical developmental stages [62].

Epigenetic modifications are central to understanding how paternal nutrition influences offspring health [63]. These modifications involve changes in histone acetylation, DNA methylation, and the function of small non-coding RNAs (sncRNAs), all of which regulate gene expression without altering the DNA sequence. Environmental factors like stress, diet, and toxin exposure can induce epigenetic changes in sperm, which may be transferred to offspring, influencing their development and increasing their risk of disease [64].

Paternal diet can alter patterns of DNA methylation in sperm, leading to lasting effects on gene expression in offspring. For instance, diets high in fat or deficient in essential nutrients can lead to the hypomethylation or hypermethylation of specific genes associated with metabolic processes, predisposing offspring to conditions like diabetes, obesity, and cardiovascular diseases [6]. Additionally, histone proteins, which organize DNA into chromatin, can be modified by paternal diet, affecting the accessibility of specific genes for transcription. Moreover, the paternal diet can alter the expression of sncRNAs, which regulate gene expression post-transcriptionally [65]. These epigenetic changes affect critical developmental pathways in offspring, further contributing to the risk of disease [66].

The transgenerational effects of paternal nutrition have also been investigated. Paternal diet can potentially influence not only immediate offspring but also future generations, a concept referred to as “transgenerational inheritance” [6]. Animal studies revealed that the metabolic consequences of paternal high-fat or low-protein diets can persist for several generations [14]. This finding suggests that the impact of paternal nutrition could extend beyond the first generation, influencing the health of grandchildren and great-grandchildren.

One of the most widely studied effects of poor paternal nutrition is the development of metabolic syndrome in offspring [56]. Metabolic syndrome is a group of conditions that includes obesity, insulin resistance, dyslipidemia, and hypertension. Paternal high-fat diets, along with other unbalanced diets, have been found to raise the risk of metabolic syndrome in offspring, mainly through epigenetic changes in genes that regulate metabolism and insulin sensitivity [9,67]. Moreover, paternal malnutrition or micronutrient deficiencies may elevate the risk of cancer in offspring [9,67].

Studies have shown that inadequate B vitamin intake in fathers during the preconception period elevated the risk of developing colorectal cancer in offspring [68], while other research indicates a potential link between paternal malnutrition and risk of breast cancer in female offspring [67]. Modifications in genes involved in tumor suppression and cell proliferation via epigenetics changes are suggested as underlying mechanisms [69].

Paternal diet can also influence cognitive and behavioral traits in offspring. For example, paternal high-fat diets have been associated with increased anxiety-like behaviors and impaired cognitive functions in animal models [6]. Epigenetic changes in genes involved in brain development and neurotransmitter regulation are proposed as potential mechanisms [70].

The current evidence strongly suggests that strategies to improve offspring health should also recognize the paternal diet along with the maternal diet during the preconception period as key factors. As the field of paternal nutrition continues to evolve, it will offer new opportunities for intervention and prevention aimed at improving the health of future generations.

While both parents’ nutritional status is important, it seems maternal nutrition tends to have a more direct and immediate impact on offspring health due to the direct provisioning of nutrients and the environment within the womb [71]. Paternal nutrition, though influential, affects offspring, primarily through genetic and epigenetic modifications that are less directly tied to the immediate maternal environment [72].

### 3.2. The Impact of Parental Obesity During Preconception

Children’s risk of obesity is strongly associated with the BMI trajectories of their parents. Studies that track parental BMI over time offer valuable insights into the intergenerational transmission of obesity [73].

#### 3.2.1. The Impact of Maternal Obesity

Maternal Body Mass Index (BMI) has been increasingly recognized for its significant influence on both physical and psychological outcomes in children [74]. Maternal obesity has been linked to adverse changes in offspring cardiovascular structure and function, including increased risk of hypertension and altered heart structure [75].

In one study [44], children born to mothers with preconception obesity showed lower resilience scores. This suggests that maternal obesity may interfere with fetal neurodevelopment through negative intrauterine conditions, such as inflammation and metabolic disturbances, which ultimately affect the child’s ability to handle stress and adversity. Conversely, children of underweight mothers showed reduced prosocial behaviors. This could be attributed to the impaired development of the neural circuits involved in social functioning due to maternal malnutrition and potential negative influences on parenting practices [44]. Higher maternal adiposity before and during pregnancy has been linked to an increased risk of neuropsychiatric disorders in offspring, such as Attention-Deficit/Hyperactivity Disorder (ADHD) and Autism Spectrum Disorder (ASD) [76]. Moreover, children born to mothers with higher adiposity levels may exhibit more behavioral problems, including difficulties with impulse control and social interactions [77,78,79,80,81,82].

The impact of both maternal and paternal obesity together during preconception has also been studied. Offspring with two obese parents showed increased hypothalamic inflammation, which is associated with disrupted energy balance and higher risk of obesity. Altered leptin signaling pathways, which are crucial for appetite regulation, were more evident in offspring with two obese parents. This led to higher food intake and obesity rates compared to the offspring of single obese parents [73,83].

Obesity during the preconception period can also impair fertility by affecting hormonal balance and ovulation [84]. Additionally, women with obesity face a higher risk of complications such as gestational diabetes, preeclampsia, and an increased likelihood of cesarean delivery [85].

#### 3.2.2. The Impact of Paternal Obesity

There is growing evidence pointing to the significant role of paternal obesity on the health and metabolic profiles of their offspring. Current research has shown that paternal obesity plays a critical role in shaping offspring’s metabolic outcomes independent of maternal weight status in the preconception period [86]. Both human and animal studies have shown that paternal obesity can alter offspring metabolism through mechanisms such as changes in sperm quality, epigenetic programming, and disruptions in metabolic pathways [87,88,89,90].

Animal studies have provided critical insights into how paternal obesity affects offspring. Rodent models, in which obesity is induced through high-fat diets, have been pivotal in elucidating the mechanisms by which paternal obesity alters offspring metabolism [91,92,93]. In another study, the offspring of obese male rodents exhibited insulin resistance, impaired glucose tolerance, and increased adiposity [94]. These disruptions mirror early-stage metabolic syndrome and potential transgenerational transmission of obesity-related conditions. Paternal obesity also adversely affects sperm quality by reducing sperm motility and concentration [95]. More importantly, paternal obesity results in epigenetic modifications that alter the developmental programming of the offspring [8]. Obesity affects the genes involved in energy balance, fat storage, lipid metabolism, and insulin signaling, suggesting that the environment during sperm development is crucial for long-term health outcomes in offspring [96]. These changes create a foundation for heightened susceptibility to obesity and metabolic diseases in later life [97].

Recent studies suggest that the impact of paternal obesity may be more profound and lasting than previously thought [98,99,100]. For instance, children of obese fathers show early signs of metabolic dysfunction, including altered lipid profiles and insulin resistance, conditions that are predictive of future metabolic diseases [101].

Both animal and human studies have highlighted several mechanisms through which paternal obesity influences offspring health. Epigenetic modifications in the sperm of obese fathers lead to altered gene expression in their offspring, particularly in pathways regulating adiposity and metabolic function. This reprogramming sets the stage for lifelong susceptibility to obesity and related disorders [96].

Beyond the immediate offspring, recent animal studies have demonstrated that paternal obesity potentially affects multiple generations, with grandchildren showing similar metabolic disturbances, such as altered glucose metabolism and increased adiposity, despite being conceived by non-obese parents [102]. This intergenerational transmission is likely driven by epigenetic changes in germ cells, indicating that lifestyle factors in one generation can have significant, lasting impacts on the health outcomes of future generations. Importantly, these findings imply that paternal preconception health is just as critical as maternal health, reinforcing the need for comprehensive strategies to address both parental roles in preconception care [103].

Emerging research also examines the potential reversibility of the metabolic outcomes of paternal obesity on offspring through dietary and lifestyle interventions. Studies have demonstrated that improvements in paternal diet and exercise before conception can reduce negative metabolic programming in offspring. For example, a study [8] found that switching male rodents from a high-fat diet to a low-fat, nutrient-dense diet before mating significantly improved metabolic parameters such as glucose tolerance and also reduced adiposity in their offspring. This highlights the potential for positive lifestyle changes in reversing or reducing the adverse transgenerational impacts of obesity [6]. Moreover, the incorporation of specific nutrients, including omega-3 fatty acids, into the paternal diet has been linked to improved sperm quality and a reduction in metabolic risks in offspring [104]. Such findings emphasize the potential for targeted dietary interventions in preconception health programs aimed at both parents, which could help in lowering the intergenerational transmission of obesity-related diseases [105].

Paternal obesity may also affect metabolic pathways in offspring, including insulin signaling, energy homeostasis, and lipid metabolism. These disruptions predispose offspring to hyperphagia (excessive eating), weight gain, and obesity-related metabolic dysfunctions such as insulin resistance [101]. Further evidence highlights how paternal obesity may compromise metabolic regulation in offspring, affecting pathways that control insulin signaling, energy balance, and lipid metabolism. These disruptions can result in hyperphagia (excessive eating), weight gain, and later metabolic dysfunctions, including insulin resistance and Non-Alcoholic Fatty Liver Disease (NAFLD) in offspring [106]. In rodent models, paternal obesity has been linked to increased oxidative stress and inflammation in offspring, which further aggravates the development of metabolic syndrome [5].

Interestingly, sex-specific differences in metabolic outcomes are being observed. The female offspring of obese fathers may have a heightened risk of reproductive and metabolic disorders, such as Polycystic Ovary Syndrome (PCOS), while male offspring tend to display a higher propensity for obesity and insulin resistance [107]. This suggests that paternal obesity may interact with offspring sex to influence the risk of specific metabolic and reproductive conditions, highlighting the complexity of paternal effects on offspring health [108].

Epigenetic modifications, including histone modifications, DNA methylation, and non-coding RNAs, have been recognized as key mechanisms driving these effects. Paternal obesity alters these epigenetic marks in sperm, which are passed onto the zygote and affect gene expression in the developing fetus [109,110]. Notably, the Insulin-like Growth Factor (IGF) pathway, an important player in regulating growth and metabolism, is particularly affected by these epigenetic changes, increasing susceptibility to metabolic disorders in the offspring [111]. Studies exploring therapeutic interventions indicate that epigenetic reprogramming may provide a means to reverse the adverse effects of paternal obesity. For instance, compounds such as resveratrol and metformin, known for their antioxidant and insulin-sensitizing properties, have been shown to reverse some of the metabolic and epigenetic alterations in the offspring of obese fathers [6,112]. This opens the possibility of using epigenetic therapies alongside lifestyle interventions to mitigate the transgenerational impact of paternal obesity.

Obesity in fathers can also lead to changes in the microRNA profile of sperm. MicroRNAs are small non-coding RNAs that are involved in the regulation of gene expression and can impact various biological processes, including those related to metabolism and development [113]. These altered sperm microRNAs can influence the health and development of offspring, contributing to conditions such as metabolic syndrome.

### 3.3. The Impact of Parental Diabetes During Preconception

Parental diabetes during preconception can significantly impact the health of offspring. Either paternal or maternal diabetes before conception can alter the metabolic environment during crucial stages of gamete formation and early embryonic development [56,106]. Hyperglycemia induced by maternal diabetes can influence the uterine environment, increasing the risk of congenital malformations, fetal growth abnormalities, and metabolic disorders in the child [114,115]. There are only a few studies available focusing on paternal diabetes. Current studies have linked paternal diabetes to epigenetic changes in sperm that may predispose offspring to obesity, insulin resistance, and cardiovascular diseases later in life [109,110].

#### 3.3.1. The Impact of Maternal Diabetes

Maternal diabetes, both pre-existing and gestational, can have profound effects on offspring, impacting fetal development and health outcomes long after birth. Hyperglycemia during pregnancy is one of the primary concerns, as it alters the intrauterine environment and exposes the fetus to elevated glucose levels. This exposure is linked to an increased risk of congenital malformations, excessive fetal growth (macrosomia), and long-term metabolic disorders in the child, including obesity, insulin resistance, and type 2 diabetes [114]. These risks are exacerbated by the fact that maternal diabetes can lead to epigenetic changes in the developing fetus, impacting gene expression related to glucose metabolism and insulin sensitivity [116].

Research indicates that maternal diabetes can disrupt normal placental function, resulting in altered nutrient transport and oxygen supply to the fetus, which in turn can affect fetal growth patterns [117]. Infants born to mothers with diabetes are at higher risk of both macrosomia and low birth weight, depending on the degree of maternal glycemic control. Macrosomia, characterized by a birth weight of more than 4000 g, is particularly concerning as it predisposes the infant to complications during delivery, such as shoulder dystocia, and increases the risk of childhood obesity [115].

In addition to immediate birth outcomes, maternal diabetes is associated with a higher risk of developing metabolic syndrome in offspring, characterized by a cluster of conditions including insulin resistance, dyslipidemia, and hypertension [118]. These metabolic alterations often emerge during adolescence and can persist into adulthood, increasing the lifetime risk of cardiovascular diseases. Studies suggest that maternal hyperglycemia during pregnancy can induce epigenetic changes in key metabolic genes, such as those involved in insulin signaling, playing a role in the development of type 2 diabetes in offspring [116].

The long-term health impacts of maternal diabetes extend beyond metabolic disorders. A growing body of evidence points to the association between maternal hyperglycemia and neurodevelopmental disorders in children. Maternal diabetes, particularly when poorly controlled, has been associated with a higher risk of Autism Spectrum Disorder (ASD) and Attention-Deficit/Hyperactivity Disorder (ADHD) in offspring [119]. These findings suggest that maternal glycemic control during pregnancy is critical not only for fetal metabolic programming but also for neurodevelopmental health.

Furthermore, maternal diabetes can contribute to altered fetal programming, where the intrauterine environment affects the fetus’s physiological development in a manner that predisposes the child to chronic diseases later in life. For instance, the offspring of mothers with diabetes exhibit higher rates of childhood obesity, which often leads to metabolic syndrome and type 2 diabetes during adulthood [114]. This process, known as the “fetal origins of adult disease” hypothesis, underscores the long-term consequences of poor maternal glycemic control on offspring health [118].

Effective management of maternal diabetes is critical for mitigating these risks. Maintaining optimal glycemic control before and during pregnancy through diet, exercise, and pharmacological interventions, when necessary, can significantly reduce the likelihood of adverse outcomes in offspring. Early identification and treatment of gestational diabetes, as well as proper follow-up postpartum, are essential to improve maternal health and, subsequently, child health [115].

#### 3.3.2. The Impact of Paternal Diabetes

Paternal diabetes has been identified as a significant risk factor for the development of metabolic disorders in offspring [120]. Children of fathers with diabetes are at an increased risk of obesity, type 2 diabetes, and insulin resistance [120], which is aligned with the growing body of evidence indicating paternal health influences offspring outcomes through genetic, epigenetic, and environmental mechanisms [121].

One of the primary concerns is the increased susceptibility of offspring to metabolic disorders, which can manifest early in life or develop progressively into adulthood. The long-term health consequences often include insulin resistance, hypertension, dyslipidemia, and even cardiovascular disease. These conditions can be worsened by lifestyle factors such as poor diet, physical inactivity, and stress, which may also be shaped by paternal behavior and other environmental influences.

Children of fathers with metabolic syndrome have been shown to exhibit a higher tendency for similar conditions, often starting with elevated BMI and progressing to insulin resistance and hypertension [120]. Various mechanisms have been suggested to explain this transgenerational effect. Genetic inheritance plays a crucial role, but epigenetic modifications may also contribute to the development of metabolic syndrome in offspring [122]. These epigenetic changes may alter the expression of the genes involved in metabolic regulation, thereby increasing the risk of metabolic disorders. Similarly, another study found that paternal diabetes was linked to a higher prevalence of pre-diabetic phenotypes in adult offspring, including insulin resistance and impaired glucose tolerance [123].

Epigenetic modifications in sperm cells may lead to altered gene expression in offspring, potentially predisposing them to metabolic disorders. For instance, DNA methylation and histone modifications have been identified as key factors in regulating the genes involved in glucose metabolism and insulin sensitivity [124]. Moreover, these epigenetic disturbances can impair β-cell function, contributing to T2D [121]. These epigenetic changes may persist through generations, increasing the risk of developing type 2 diabetes and related metabolic conditions [121]. Additionally, poor sperm quality, often observed in men with diabetes and metabolic syndrome, may contribute to altered embryonic development, affecting fetal growth and metabolic programming [88]. This could predispose offspring to metabolic challenges from birth, such as a lower birth weight. Lower birth weight is linked to a catch-up growth phase and a higher risk of developing insulin resistance and type 2 diabetes in later life. Higher prevalence of obesity, insulin resistance, hypertension, and dyslipidemia, all of which are major risk factors for cardiovascular disease, are consequences for offspring [125].

In a study comparing children with healthy parents to those with one or both parents affected by diabetes and Metabolic Syndrome (MS), those with healthy parents exhibited significantly lower rates of diabetes and MS. Further analysis of children with one affected parent revealed that those with a father suffering from both diabetes and MS, alongside a healthy mother, had the highest levels of BMI, Systolic Blood Pressure (SBP), and total cholesterol, outpacing even children with both parents affected. This suggests that paternal dietary habits may significantly influence offspring health, even when the mother is healthy [120].

Paternal diabetes is also linked to a higher risk of low birth weight in children. An epidemiological study identified a strong genetic link between paternal diabetes and low birth weight, which subsequently increases the risk of type 2 diabetes later in life [126]. In this community, children born at low birth weight faced heightened lifetime risks of diabetes, although the authors noted difficulty in determining whether paternal diabetes was the sole contributor to low birth weight or if other environmental factors also played a role. This finding contrasts sharply with the well-established link between maternal impaired glucose tolerance and higher birth weight [127].

Additionally, paternal diabetes may lead to glucose intolerance and insulin resistance in children; ref. [125] found that offspring’s birth weight was inversely related to paternal insulin resistance and diabetes in later life. The genetic factors influencing insulin action help explain the correlation between offspring birth weight, adult cardiovascular disease, and diabetes risk. Over time, fasting glucose levels in the children of diabetic fathers have been shown to rise. The fasting glucose levels of offspring from fathers with diabetes has been shown to elevate as age progresses [121]. A study involving Pima Indians categorized children into three groups: those with a diabetic father and healthy mother (ODF), those with a diabetic mother and healthy father (OMED), and a control group with both healthy parents (CON) [123]. The results showed that ODF children had lower insulin secretion than both the OMED and CON groups. However, after adjusting for body size, ODF children demonstrated comparable insulin action. Notably, a longer insulin secretion test revealed persistent lower secretion in the ODF group. This research highlights the significant role of paternal heritability in body composition and beta-cell dysfunction, suggesting that epigenetic mechanisms may contribute to susceptibility to diabetes [123].

In another study by Wei et al. [124], paternal prediabetes was linked to glucose intolerance and insulin resistance in offspring. The negative effects were attributed to changes in gene expression in the pancreatic islets of fathers, leading to down-regulation of the key genes involved in glucose metabolism and insulin signaling, which were also observed in their offspring. This study showed that inducing paternal prediabetes in mice resulted in higher blood glucose levels and reduced insulin sensitivity in their offspring, with these effects worsening as the offspring aged [124]. Additionally, the study explored how changes in gene expression and methylation patterns in pancreatic islets can be inherited from fathers with diabetes. Researchers identified 402 genes in the pancreatic islets of mice altered by paternal prediabetes. Notably, the expression of Phosphofructokinase (PFK), an enzyme critical in the glycolytic pathway, was significantly reduced, while levels of Protein tyrosine phosphatase nonreceptor type 1 (Ptpn1), which inhibits insulin signaling, were markedly elevated. These changes likely contribute to impaired insulin signaling in the offspring of prediabetic fathers. Additionally, the study noted alterations in the methylation patterns associated with intron element genes in the pancreatic islets of offspring, further elucidating the potential mechanisms leading to diabetes in children of prediabetic fathers [124].

Lastly, paternal diabetes has been linked to negative cardiovascular outcomes in offspring, including elevated systolic blood pressure and total cholesterol. The research by Wannamethee et al. [125] indicated a genetic connection between offspring birth weight and the increased future risk of diabetes and cardiovascular disease. Fathers of lower birth weight children exhibited greater insulin resistance, higher diabetes prevalence, and increased mean blood pressure. The study even extended its analysis to the second and third children of these fathers, reaffirming the strong inverse relationship between offspring birth weight and insulin resistance.

### 3.4. The Impact of Parental Exercise During Preconception

The impact of parental exercise during the preconception period has been extensively studied, highlighting the long-term benefits of physical activity not only for parents but also for future generations. Exercise by both mothers and fathers can positively influence the metabolic health and overall well-being of their offspring. Regular physical activity during this critical period has been shown to improve gamete quality, enhance fertility, and optimize the intrauterine environment, resulting in better outcomes for the developing embryo. Additionally, exercise-induced epigenetic changes in both sperm and eggs may play a key role in shaping the long-term health of offspring, reducing the risk of conditions like obesity, diabetes, and cardiovascular disease later in life.

#### 3.4.1. The Impact of Maternal Exercise

Maternal exercise before and during pregnancy has been shown to have beneficial effects on the metabolic health of offspring [128]. Maternal exercise improves the metabolic parameters in the offspring by enhancing mitochondrial function [128,129] and reducing the risk of developing metabolic diseases such as obesity, type 2 diabetes, and cardiovascular diseases. These benefits are associated with improved placental function and the delivery of nutrients to the fetus [128]. Similarly, paternal exercise prior to conception has been shown to positively affect the metabolic health of offspring. Regular paternal exercise can improve glucose metabolism and reduce the risk of obesity in offspring by inducing beneficial changes in sperm epigenetics [130,131].

These changes include alterations in DNA methylation and non-coding RNA expression, which are associated with improved insulin sensitivity and metabolic function in offspring [131]. Building on the research surrounding parental exercise, recent studies suggest that paternal physical activity plays a crucial role in shaping offspring’s metabolic health. Regular physical exercise by the father before conception has been shown to improve their offspring’s glucose tolerance, reduce adiposity, and enhance insulin sensitivity [132,133]. These benefits are believed to arise from exercise-induced epigenetic changes, including alterations in DNA methylation, histone acetylation, and non-coding RNA expression in sperm, which influence gene expression related to metabolic function in the offspring [132,133].

#### 3.4.2. The Impact of Paternal Exercise

Paternal exercise has also been associated with improvements in cardiovascular health and reductions in inflammatory markers in offspring [113]. Notably, even modest levels of physical activity can induce beneficial epigenetic reprogramming that enhances offspring resilience against metabolic diseases such as obesity, type 2 diabetes, and cardiovascular conditions later in life [132,134].

These findings highlight the potential for targeted interventions that incorporate both parents in preconception health strategies aimed at optimizing the health of future generations. Interestingly, recent research has explored the combined impact of maternal and paternal exercise on offspring health. Studies suggest that when both parents engage in regular exercise before conception, the offspring experience additive metabolic benefits, such as improved glucose metabolism and enhanced mitochondrial function [135]. This demonstrates the synergistic effect of parental exercise, underscoring the importance of addressing both maternal and paternal preconception health in efforts to prevent the transgenerational transmission of metabolic diseases.

The combination of both maternal and paternal exercise produces an additive effect, leading to even greater improvements in offspring metabolic health. These combined effects are more robust in preventing metabolic disorders and ensuring better overall health outcomes in offspring compared to singular parental exercise [128]. Improved glucose and lipid metabolism, enhanced energy expenditure, and a reduced risk of developing metabolic disorders are among the long-term effects of parental exercise during the periconceptional period in offspring [128]. Epigenetic modifications induced by exercise play a critical role in mediating these positive effects on offspring metabolism. These changes help regulate key metabolic pathways and contribute to the long-term health benefits seen in offspring [128,131].

#### 3.4.3. Interactive Effects of Diet and Exercise

The interactive effects of exercise and maternal high-fat diet before and during pregnancy have also been studied: offspring born to mothers who consumed a high-fat diet during pregnancy were at an increased risk for metabolic disorders, including obesity, glucose intolerance, and insulin resistance [136]. These adverse effects were particularly evident in male offspring [136]. Maternal exercise, both before and during pregnancy, prevented the negative metabolic consequences of a maternal high-fat diet on offspring. Exercise improved glucose metabolism, reduced fat accumulation, and improved insulin sensitivity in male offspring, even when the mothers consumed a high-fat diet. The authors suggested that exercise before pregnancy is as important as exercise during pregnancy in safeguarding the metabolic health of offspring [136]. In another study, a paternal high-fat diet before conception negatively impacted the offspring’s metabolic health, particularly increasing the risk of insulin resistance and metabolic disorders, such as type 2 diabetes, in skeletal muscle. A four-week exercise intervention early in life (starting in the juvenile period) could “reprogram” the metabolic impairments in offspring caused by their father’s high-fat diet. This significantly improved insulin sensitivity and prevented the onset of skeletal muscle insulin resistance in adulthood. It also enhanced mitochondrial function in skeletal muscle [129]. Similarly, Larson et al. found that a paternal high-fat diet led to reduced placental and fetal tissue weights, along with decreased expression of nutrient transporter genes in the placental tissues of male offspring (F1). In contrast, paternal exercise (voluntary wheel running) for three months before mating resulted in increased placental tissue weight, enhanced expression of nutrient transporter genes, and greater fetal weights, while also reducing placental inflammatory gene expression [137]. In mice, both maternal and paternal exercise improved glucose tolerance in offspring at 52 weeks of age. Offspring from exercising parents (maternal, paternal, or both) showed lower blood glucose levels and better insulin sensitivity compared to those from sedentary parents. Parental exercise also led to changes in the offspring’s pancreatic function, particularly in the size and mass of beta cells. While individual maternal or paternal exercise reduced beta cell size, the combined exercise of both parents had the most pronounced effects, decreasing both beta cell and islet size and improving pancreatic health [135]. In a study with seven-week-old male C57BL/6 mice, those fed a high-fat diet showed impaired glucose tolerance and increased fat mass in their offspring by 52 weeks. However, paternal exercise improved glucose tolerance, reduced fat mass, and enhanced glucose uptake in the offspring. The researchers suggested that these benefits were linked to changes in sperm physiology, such as improved sperm motility and altered small RNA profiles. Paternal exercise led to significant metabolic health improvements in the offspring [138]. Similarly, the offspring of male mice that underwent exercise training displayed improved glucose homeostasis and insulin sensitivity. Paternal exercise altered DNA methylation patterns of the PI3Kca gene in the offspring’s skeletal muscle. Interestingly, similar DNA methylation changes were observed in the sperm of exercised males, suggesting that exercise-induced epigenetic modifications during germ cell development contribute to these transgenerational effects [139]. In another study, adult male Wistar rats were divided into sedentary and exercised groups. While physical parameters, cognitive performance, and BDNF expression were similar in the male offspring, those of exercised fathers had lower gonadal fat and reduced global DNA methylation in the hippocampus compared to those of sedentary fathers. The authors suggested that paternal exercise affects offspring adiposity and hippocampal epigenetic programming, emphasizing the potential benefits of physical activity [140]. Similarly, in another rat study, the offspring of exercised fathers showed improved spatial learning and a significant decrease in hippocampal global DNA methylation levels compared to the offspring of sedentary fathers [140].

As previously stated, obesity in fathers can lead to changes in the microRNA profile of sperm. MicroRNAs are small non-coding RNAs that regulate gene expression and can influence various biological processes, including those related to metabolism and development [113]. After 8 weeks of diet or exercise interventions in obese founder male mice, restored insulin sensitivity and normalized adiposity in female offspring was observed. It also corrected the levels of X-linked sperm microRNAs that influence the genes involved in cell cycle and apoptosis, crucial for oocyte and early embryogenesis. Additionally, obesity-related issues like inflammation and glucose intolerance were linked to changes in sperm microRNA levels and offspring health. Thus, improving paternal metabolic health before conception can partially reverse abnormal epigenetic signals in sperm and enhance the metabolic health of female offspring [113]. In another study on mice, paternal exercise before conception showed protective effects against increased T2D risk in male offspring resulting from a paternal HF diet by improving insulin signaling in skeletal muscle [141]. Similarly, another study found that paternal exercise reversed the negative effects of a high-fat diet on offspring mice, improving glucose tolerance, fat mass, and glucose uptake in skeletal muscles. This was linked to exercise-induced changes in small RNAs, which helped mitigate the sperm RNA alterations caused by a high-fat diet [138].

Parental physical exercise during preconception has emerged as a significant factor influencing offspring health through various epigenetic and metabolic pathways. While immediate effects on cognitive and physical development may not always be apparent, the long-term benefits of parental exercise on metabolic health and disease prevention are promising. Continued research in this field is crucial for fully understanding the mechanisms involved and for developing targeted interventions to enhance the health of future generations. The synergistic effects of maternal and paternal exercise have been shown in various studies, indicating a more robust effect on preventing metabolic disorders and ensuring better overall health outcomes in offspring compared to singular parental exercise alone.

## 4. The Impact of Parental Diet on Metabolism

The parental diet during preconception period has a significant impact on the metabolism and immune system of offspring, influencing health outcomes that extend across their lifespan. Additionally, parental diet during preconception influences immune system development in the offspring.

### 4.1. The Effect of Parental Diet on Glucose Metabolism in Offspring

There is growing evidence supporting the role of both maternal and paternal diets in the regulation of glucose metabolism in offspring.

#### 4.1.1. The Impact of Maternal Diet

The maternal diet has a profound impact on glucose metabolism in offspring, influencing fetal development and long-term metabolic health. Studies have shown that maternal overnutrition, especially a high-fat or high-sugar diet during pregnancy, can increase the risk of glucose intolerance, insulin resistance, and obesity in offspring. One study found that the offspring of obese mothers on a high-fat diet had impaired glucose tolerance, hyperinsulinemia, and higher fat mass, caused by disrupted insulin signaling and β-cell function in the pancreas [142]. These effects persisted into adulthood, increasing the offspring’s risk for developing metabolic syndrome and type 2 diabetes [142].

Maternal obesity and a high-fat diet during pregnancy are linked to a higher risk of glucose intolerance and insulin resistance in offspring. Maternal overnutrition can lead to excessive fetal nutrient exposure, resulting in altered pancreatic development, impaired insulin sensitivity, and an increased risk of metabolic syndrome in later life [143]. Furthermore, a maternal diet not only affects glucose metabolism through direct nutrient exposure but also via epigenetic mechanisms that influence gene expression. A maternal high-fat diet has been shown to result in the downregulation of the key genes involved in glucose metabolism, including the genes responsible for insulin sensitivity and glucose transport in tissues such as the liver and skeletal muscles [135]. This epigenetic programming can result in long-term metabolic dysfunction in offspring, even without exposure to a high-fat diet after birth.

In contrast, maternal undernutrition can also disrupt glucose. One study [144] found that maternal protein restriction during pregnancy impaired fetal β-cell development, reducing insulin production and glucose tolerance in offspring. This was accompanied by smaller pancreatic islets and reduced insulin gene expression, leading to impaired glucose metabolism and an increased risk of type 2 diabetes and other metabolic diseases later in life. Additionally, a low-protein maternal diet has been associated with reduced fetal β-cell mass and impaired insulin production, raising the risk of glucose intolerance and diabetes in adulthood [145]. Maternal malnutrition also affects epigenetic programming, altering the genes involved in glucose and lipid metabolism, with potential long-lasting effects into adulthood [146].

Micronutrient deficiencies during pregnancy also play a critical role in shaping offspring glucose metabolism. For example, maternal folate deficiency has been associated with impaired glucose homeostasis and increased adiposity in offspring, possibly through disruptions in DNA methylation and other epigenetic modifications [147]. Ensuring adequate maternal nutrition, including sufficient micronutrient intake, is crucial for supporting healthy metabolic outcomes in offspring.

#### 4.1.2. The Impact of Paternal Diet

The paternal diet plays a significant role in regulating glucose metabolism in offspring. A study examined the effects of a Low-Protein Diet (LPD) on semen quality and the health of offspring. Male mice on an LPD for 8 weeks showed hypomethylation and reduced expression of DNA methylation and folate-cycle regulators in the testes. The offspring of these males exhibited increased adiposity, glucose intolerance, nonalcoholic fatty liver disease symptoms, and altered gut microbiota. However, the LPD did not affect testis weight, sperm count, pregnancy rates, or litter size. Females mated with LPD males had impaired uterine immunological signaling and vascular remodeling [148].

Conversely, paternal exercise has been shown to enhance glucose metabolism in adult offspring. In one study, seven-week-old male mice were fed either a chow diet or a high-fat diet while being in cages with running wheels. After three weeks, sperm from one group of male mice was used to breed sedentary females on a chow diet. The offspring, also fed chow and kept sedentary, were monitored for their first year of life. The findings indicated that paternal exercise mitigated the negative effects of a high-fat diet, reversing impairments in glucose tolerance, fat mass percentage, and glucose uptake in skeletal muscles [138].

Another study focused on the impact of a high-fat paternal diet on offspring. Male mice were given ad libitum access to water and assigned to either a control diet (6% butter fat) or a high-fat diet (21% butter fat) from 5 to 17 weeks. They were then mated with eight-week-old normal females to produce offspring in the final week of the diet. Offspring from high-fat diet males showed increased body weight and adiposity. These offspring had higher serum cholesterol, triglyceride, and NEFA levels compared to control-fed littermates. High-fat feeding prior to mating led to impaired glucose tolerance and insulin secretion in female offspring, linked to reduced pancreatic islet size and gene expression changes. The diets did not affect the mass of muscles, pancreas, or kidneys in male offspring [149].

Overall, both paternal and maternal diets play critical roles in shaping glucose metabolism in offspring, and improving preconception and prenatal nutrition may mitigate the risk of metabolic disorders in future generations.

### 4.2. The Effect of Parental Diet on Lipid Metabolism in Offspring

Although research in this area remains limited, emerging studies have begun to investigate the influence of both paternal and maternal diets on lipid metabolism in offspring.

#### 4.2.1. The Impact of Maternal Diet

Maternal diet plays a critical role in shaping lipid metabolism in offspring, with maternal high-fat diets particularly influencing metabolic health. Studies show that maternal obesity and high-fat diet consumption during pregnancy can lead to increased lipid accumulation in the offspring’s liver, increasing their risk of developing Non-Alcoholic Fatty Liver Disease (NAFLD) [150], dyslipidemia, and obesity later in life [142]. These lipid changes were linked to alterations in gene expression related to lipid metabolism, including the increased expression of the genes involved in lipogenesis and decreased expression of the genes responsible for fatty acid oxidation.

Maternal overnutrition can result in fetal overexposure to lipids, leading to changes in fetal lipid metabolism, fat storage, and adipocyte differentiation [151]. This disruption in lipid metabolism can continue into adulthood, raising the offspring’s risk of developing metabolic disorders like cardiovascular disease and type 2 diabetes.

Another study explored the effects of maternal omega-3 Polyunsaturated Fatty Acid (PUFA) supplementation during pregnancy on lipid metabolism in offspring. It found that omega-3 supplementation helped reduce triglyceride levels and improved lipid profiles in the offspring, compared to a control group. The study suggested that maternal intake of omega-3 PUFAs may modulate gene expression related to lipid transport and storage, thus preventing dyslipidemia in offspring [152].

Furthermore, maternal undernutrition has been shown to have long-term consequences on lipid metabolism in offspring. One study [153] found that maternal protein restriction during pregnancy led to the dysregulation of lipid metabolism in the liver of rat offspring. The study revealed that the offspring of protein-restricted mothers had reduced levels of the key enzymes involved in lipid oxidation and an increased accumulation of triglycerides in the liver, potentially contributing to metabolic syndrome development in adulthood. Another study [154] demonstrated that maternal protein restriction during pregnancy resulted in higher plasma cholesterol and triglyceride levels in adult offspring, along with altered hepatic lipid metabolism. The findings emphasized that maternal malnutrition could induce long-lasting changes in lipid metabolism, increasing the risk of cardiovascular diseases in later life.

Epigenetic modifications, such as DNA methylation, have also been implicated as mediators of the impact of maternal diet on lipid metabolism. One study [155] showed that maternal high-fat diets induced hypermethylation of the genes involved in fatty acid oxidation and lipolysis in the liver of offspring, contributing to lipid accumulation and obesity.

These findings underscore the importance of maternal diet in regulating offspring lipid metabolism and suggest that interventions targeting maternal nutrition during pregnancy could help mitigate the risk of metabolic disorders in offspring.

Maternal micronutrient deficiencies can also have a lasting impact on lipid metabolism in offspring. One study [147] found that maternal folate deficiency was associated with increased fat mass and altered lipid metabolism in the offspring, highlighting the importance of adequate micronutrient intake during pregnancy. The study suggested that folate deficiency during pregnancy may alter lipid metabolism through epigenetic modifications that persist into adulthood, increasing the risk of obesity and metabolic disorders.

#### 4.2.2. The Impact of Paternal Diet

In one study, the impact of preconception paternal vitamin intake on intestinal tumor formation, hepatic gene expression, and lipid content in female offspring was investigated. Male mice, genetically predisposed to intestinal tumors, were fed one of three diets: control; mildly deficient; or supplemented with vitamins B2, B6, and B12 and folate over an 8-week period before mating with wild-type females on a standard control diet [68]. The study found no significant differences in blood insulin and glucose levels among the groups. However, the mice on the mildly deficient diet exhibited modestly lower body weights compared to those on the control diet, with a 10.1% reduction in weight by week 9. The offspring of fathers on the supplemented and mildly deficient diets also had significantly lower body weights than those from control fathers [68]. Additionally, hepatic triglycerides and cholesterol levels were three times higher in the adult female offspring of fathers on the supplemented diet. Although paternal diet did not influence tumor incidence, the size of tumors varied between groups in a sex-specific manner. Both paternal and maternal diets play critical roles in shaping lipid metabolism in offspring. While paternal preconception vitamin intake has been shown to alter hepatic lipid content and gene expression in offspring, maternal high-fat diets, undernutrition, and micronutrient deficiencies significantly impact offspring lipid metabolism, potentially increasing the risk of metabolic diseases.

## 5. The Impact of Parental Diet on Immune System and Inflammation

Parental diet during the preconception period plays a critical role in shaping the immune health of offspring, with lasting impacts on inflammation and also on disease susceptibility. The effect of parental diets and specific nutrients such as anti-inflammatory diets high in omega-3 fatty acids, antioxidants, and vitamins (such as vitamins D, A, and C), and conversely, diets high in refined sugars, trans fats, and processed foods that may trigger pro-inflammatory responses and potentially alter immune system development, has been investigated. Research underscores the importance of balanced parental nutrition for fostering resilience against inflammation in offspring.

### 5.1. The Impact of Parental Health on the Immune System

Parental health before conception plays a key role in shaping the immune system of offspring, affecting their vulnerability to infections, allergies, and chronic diseases. Factors such as parental nutrition, stress, and exposure to toxins during this period can influence the immune system in offspring, often through epigenetic changes that modify gene expression related to immunity.

#### 5.1.1. The Effect of Maternal Diet

The maternal diet during pregnancy plays a pivotal role in shaping the immune system of the offspring, often exerting a greater influence than paternal factors. Studies have shown that maternal nutrition affects fetal immune system development through nutrient availability, epigenetic regulation, and inflammatory processes. For example, maternal undernutrition or malnutrition during pregnancy can alter thymic development, resulting in an impaired immune system and increased susceptibility [156].

A maternal high-fat diet has been linked to increased inflammation in both the mother and the developing fetus. In animal studies, mothers on high-fat diets experienced heightened levels of pro-inflammatory cytokines, which in turn led to altered immune cell function in the offspring [157]. These immune changes included a decrease in regulatory T cells, which are crucial for maintaining immune tolerance and preventing autoimmune diseases. Additionally, maternal obesity has been linked to impaired fetal immune responses, predisposing offspring to immune dysregulation and a heightened risk for conditions such as asthma and allergies later in life [158].

Maternal micronutrient deficiencies, such as an insufficient intake of vitamin D, have also been shown to compromise offspring immunity. Low maternal vitamin D levels during pregnancy have been linked to an increased risk of respiratory infections and autoimmune disorders in offspring [159]. Vitamin D is crucial for the differentiation and function of immune cells, including T lymphocytes and macrophages, highlighting its role in prenatal immune programming.

Moreover, maternal consumption of omega-3 fatty acids has been shown to have protective effects on the immune system of offspring. One study [160] demonstrated that maternal omega-3 supplementation during pregnancy enhanced immune cell function in the offspring, particularly in the regulation of inflammatory responses. The study found that the offspring of mothers who received omega-3 supplements exhibited increased levels of anti-inflammatory cytokines and a reduced risk of immune-mediated diseases, such as asthma.

#### 5.1.2. The Effect of Paternal Diet

Research on how parental diet may influence immune epigenetic changes in offspring is currently limited, though several studies highlight the need for further investigation. On the paternal side, epigenetic modifications are crucial for immune system regulation, affecting processes like lymphocyte differentiation and immune responses. Studies in nutri-epigenetics have shown that paternal diet impacts DNA methylation and histone modification in both somatic tissues and germline, potentially transmitting effects to offspring. A study evaluated the impact of parental immune status by subjecting mothers and fathers to immune challenges with live bacteria or a control solution, creating four treatment groups where one, neither, or both parents were challenged. Offspring from these families were raised on a food-restricted diet. Offspring from immune-challenged mothers exhibited enhanced immune responses, suggesting a form of immune priming passed from mother to offspring while the effects of paternal immune challenge were less pronounced compared to maternal challenges [161].

In another study, the impact of dietary Astragalus Polysaccharides (APS), immune modulators found in epithelial cells, on animal immunity was examined. In breeder cocks supplemented with 10 g/kg APS, paternal IL-4 levels were significantly down-regulated, while Interferon-α (IFN-α) and Interferon-β (IFN-β) levels remained unchanged. Interestingly, the offspring of these cocks exhibited significantly increased serum IFN-α and IFN-β levels, although serum IL-4 levels were unaffected. Chronic APS supplementation did not alter gene transcription in the spleen of either the breeder cocks or their offspring, particularly concerning endotoxin tolerance-related regulators [162]. The study observed several improvements in spleen immunity in the offspring, including enhanced lymphocyte proliferation and increased levels of immune-related cytokines. These findings indicate a bolstered immune response and better overall immune function. The authors suggested that the positive effects on offspring immunity might be due to epigenetic changes induced by the Astragalus polysaccharides in the paternal diet.

Another study involving two populations of beetle aimed to compare survival, immunity, and genetic outcomes to assess how paternal immune priming can be transmitted. The beetles underwent immune priming treatments before mating. The offspring were then challenged with live bacteria. The study measured the activity of Phenoloxidase (PO), an important enzyme in insect immunity. The offspring of immune-primed males displayed significantly higher PO activity and improved survival rates when exposed to live bacteria compared to those from non-primed males. However, this study did not explore genetic alterations that might establish a causal link between epigenetic changes and transgenerational immunity. Researchers observed paternal transgenerational immune priming in red flour beetles (*Tribolium castaneum*), where fathers exposed to the insect pathogen Bacillus thuringiensis conferred immune protection to their offspring. In contrast, step-offspring, who did not share genetic material with the primed father, did not show similar improvements in immune function [162].

A human study examined the influence of paternal weight on inflammation and altered immunoglobulin profiles in neonates. By analyzing vital records and paternal BMI, researchers assessed pro-inflammatory biomarkers and cytokines in newborns. A study categorizing paternal BMI into underweight, normal weight, overweight, and obese found no significant link between paternal overweight/obesity and neonatal inflammation (CRP levels). However, neonatal IgM levels were notably lower in the offspring of overweight and obese fathers, with no other significant associations observed between Ig levels and paternal obesity [163].

### 5.2. The Impact of Parental Health on Inflammation

The effect of body weight status of parents on offspring inflammatory markers is also studied in humans. One study found that parental obesity is associated with increased levels of inflammatory markers in neonates. Maternal BMI categories of overweight were associated with increased neonatal inflammation scores. However, no increase in the obese class I group was observed. This suggests that obesity in parents can influence the inflammatory state of their newborns [163].

A study on paternal obesity using high-fat-diet-fed mice examined four mating groups: lean mothers with lean or obese fathers, and obese mothers with lean or obese fathers. Paternal obesity alone did not influence offspring food intake or leptin levels, while maternal obesity was linked to hyperphagia and higher leptin levels. However, paternal obesity was associated with increased inflammatory markers, including IL-6 and TNF-alpha, in offspring [164]. Researchers explored the impact of paternal sepsis on sperm methylation and offspring immune responses. Sepsis was induced in male mice via Cecal Ligation and Puncture (CLP), and these males were then mated with healthy females. Analysis revealed altered sperm morphology, motility, and global DNA methylation in septic males. When the offspring’s immune responses were stimulated, only male descendants of post-septic fathers exhibited a reduced systemic and pulmonary immune response [165]. In another study, the effects of a Low-Protein Diet (LPD) in males on inflammation in their offspring was investigated. Mice on a Normal-Protein Diet (NPD) consumed 18% casein, while LPD males were fed a diet containing 9% casein. After seven weeks on these diets, the LPD males were mated with NPD females. Only the male offspring of LPD fathers experienced significant increases in inguinal and total fat by 24 weeks. Furthermore, genetic expression analysis of heart tissue from these LPD offspring revealed notable decreases in adenylate cyclase 5, phospholipase C β1, and protein kinase C β [37].

## 6. The Impact of Parental Diet on Offspring Behavior

The influence of parental diet on offspring extends beyond physical health, shaping the behavioral and cognitive landscape of the next generation. Emerging research has revealed that parental diet, encompassing both maternal and paternal nutrition, is pivotal in shaping the behavioral, cognitive, and psychological outcomes in offspring. While the maternal diet has long been highlighted for its direct influence on fetal development, increasing evidence underscores the role of paternal diet in transmitting epigenetic information through sperm.

### 6.1. The Impact of Maternal Diet

Maternal nutrition during preconception, pregnancy, and lactation plays a fundamental role in offspring neurodevelopment, affecting behavioral and emotional outcomes well into adulthood [166]. Nutritional programming, where a mother’s diet shapes her child’s development, is primarily driven by epigenetic mechanisms like DNA methylation and histone modification [167]. Essential nutrients, including folic acid, B vitamins, and essential fatty acids, are crucial for fetal brain development and help reduce the risk of neurodevelopmental disorders [168]. Inadequate maternal folate, for example, has been associated with cognitive deficits and behavioral irregularities in offspring, as insufficient levels disrupt normal methylation patterns crucial for cognitive function [169,170]. However, excess folate supplementation during pregnancy leads to increased anxiety and hyperactivity, as demonstrated in animal studies [171,172]. This underscores the long-term impact of maternal diet on offspring behavior and the preventive potential of ensuring sufficient maternal nutrient intake.

Maternal high-fat and high-sugar diets are linked to negative behavioral outcomes in offspring, including higher risks of attention problems, hyperactivity, and anxiety [173,174]. Diets rich in fats and sugars have been shown to disrupt the reward system through alterations in dopamine signaling pathways, essential for regulating mood and behavior [175,176]. In rodent studies, the offspring of mothers with high-fat diets exhibit hyperactivity and memory deficits, providing a model for the effects observed in human children [177,178]. Studies in humans similarly indicate that children born to mothers with high-sugar diets often struggle with emotional control and behavior problems [179,180]. These associations emphasize the importance of dietary balance during pregnancy, as high-fat and high-sugar intake can interfere with neurodevelopmental processes essential to behavioral health.

Omega-3 fatty acids, particularly DHA, play a contrastingly protective role in neurodevelopment, with maternal intake positively linked to cognitive and emotional resilience in offspring. Studies indicate that children whose mothers consumed adequate Omega-3s demonstrate better social behavior, memory function, and stress resilience [181,182]. Animal studies have demonstrated that Omega-3s induce a decrease in the parameters of anxiety, improve memory, and are essential for neurotransmitter function, further supporting their benefits for behavioral health [183,184,185]. Low omega-3 levels and an imbalance between omega-6 and omega-3 in maternal diets are associated with abnormal brain development and heightened anxiety-related behaviors in offspring, emphasizing the need for balanced maternal nutrition [186,187,188].

In addition to macronutrients, maternal micronutrient deficiencies, specifically in iron and vitamin D, have been associated with neurodevelopmental delays and increased risk for behavioral disorders. Iron deficiency, for example, disrupts myelination and neurotransmitter balance, which are crucial for attention and emotional regulation [189]. Studies show that iron-deficient mothers are more likely to have offspring with attention issues, ADHD, and other cognitive challenges [190,191]. Maternal vitamin D intake is another nutrient that has been reviewed as it relates to neurological diseases in offspring. Vitamin D deficiency in mothers has been linked to alterations in memory and schizophrenic tendencies in offspring later in life [192,193,194,195]. These findings further underscore the significant role of maternal nutrition in shaping the behavioral health of offspring.

### 6.2. The Impact of Paternal Diet

While maternal diet has traditionally received more focus, a growing body of evidence has revealed that paternal nutrition plays an equally impactful role in offspring behavior [196]. Unlike maternal diet, which impacts offspring through direct nutritional supply, paternal diet affects offspring behavior primarily through epigenetic transmission. This occurs as nutritional deficiencies, excesses, and other dietary factors can alter DNA methylation patterns in sperm, impacting gene expression in ways that influence offspring’s behavior and cognitive outcomes [6].

Folate has been extensively reviewed as it relates to maternal intake and fetal development as deficiencies are associated with neural tube defects [197]. Emerging evidence suggests that paternal folate intake also influences offspring development and behavior. A rodent study found that the offspring of rats with paternal folate deficiency were more prone to anxious and depressive traits [25]. Paternal folate supplementation has been shown to influence offspring cognitive and neural function, likely through altered methylation patterns that affect neural development, impacting learning and memory [198]. These findings point to the critical influence of paternal diet on the developing neuroendocrine system, emphasizing that both parents’ diets are crucial for fostering healthy offspring behavior.

Further, high-fat diets in fathers are associated with increased risks for metabolic dysregulation in offspring, which in turn impacts cognitive and emotional development. Animal studies demonstrate that paternal intake of high-fat diets induces increased anxiety-like behavior [88,199]. High-fat paternal diets are also linked to the inheritance of inflammatory markers, which interfere with offspring neurodevelopment, potentially leading to issues like anxiety and mood dysregulation [200]. These studies suggest that fathers’ dietary patterns before conception can shape their offspring’s behavioral outcomes in significant ways, underscoring the broader impact of a balanced diet for both parents.

In addition, paternal substance use, particularly alcohol, has been shown to cause early developmental delays, cognitive impairment, aggressive behavior, and anxiety-like behavior in offspring [201,202,203]. Alcohol intake in fathers induces epigenetic changes in sperm that can disrupt brain development in offspring, influencing stress response and mood stability [204,205,206]. These findings indicate that paternal lifestyle choices, including moderate alcohol consumption, play a role in shaping offspring behavior, reinforcing the need for prospective fathers to consider dietary and lifestyle modifications before conception.

Similar to the mechanisms proposed for alcohol-induced changes, paternal exposure to endocrine-disrupting chemicals, particularly Bisphenol A (BPA), reveals notable effects on offspring behavior. BPA, known for its estrogenic activity, can disrupt hormonal pathways and induce epigenetic modifications in the paternal germline, leading to developmental and behavioral changes in offspring [207]. Studies show that paternal BPA exposure is linked to behavioral changes in offspring, including increased anxiety, impaired social interactions, and cognitive deficits, often due to disrupted gene expression related to neural development, stress response, and metabolic pathways [207,208,209]. These findings highlight the potential for paternal BPA exposure to cause long-term, transgenerational effects on mental health and cognitive function in offspring, emphasizing the role of environmental and lifestyle factors in shaping neurodevelopmental outcomes.

## 7. Future Directions

Future research on the influence of parental nutrition, body weight, and lifestyle on offspring health should prioritize human studies, validating the applicability of findings in diverse populations. A critical area could be examining the epigenetic pathways through which specific paternal and maternal preconception factors, including micronutrient intake, antioxidant levels, and balanced macro- and micro-nutrients, affect gene expression in offspring. For example, for parental folate, vitamin D, iron, and iodine deficiencies on offspring health outcomes, future studies may focus on the long-term tracking of children born to parents undergoing targeted supplementation and dietary adjustments. Additionally, the role of balanced caloric intake and physical activity as modifiers of genetic and metabolic outcomes in offspring should be explored in more detail.

Another promising direction can be the transgenerational effects of preconception health, as they highlight the potential influence of parents’ diets on multiple generations. The focus can be on epigenetic changes in sperm and oocytes and how they might be influenced by parental nutrition and lifestyle choices that can be inherited by multiple generations. Investigating the potential synergic or opposite effects of lifestyle factors, including physical activity, stress, and nutritional influences, could provide valuable insights into how offspring health could be promoted through combined interventions. Interventions involving physical exercise for both parents before conception have shown positive effects on offspring metabolic health, including improved glucose tolerance and reduced fat mass, suggesting that preconception wellness initiatives that target both diet and exercise could have amplified benefits.

Furthermore, a more holistic approach incorporating both maternal and paternal contributions to offspring health could lead to better-informed public health policies. This may involve preconception health programs that advocate for nutritional counseling, physical fitness, and stress management as standard care for prospective parents during the preconception period. Understanding how the interplay of diet, exercise, and mental well-being can mitigate risks for metabolic syndrome across generations may prove to be a transformative approach to combatting the rise of chronic metabolic conditions in future populations.

## 8. Study Limitations and Strengths

This manuscript has several strengths, including a comprehensive review of various types of study and a novel focus on paternal health, specifically its role in nutrition and epigenetics, alongside maternal factors. It integrates epigenetic mechanisms to provide a deeper understanding of how parental health affects offspring outcomes and examines a wide range of preconception factors, offering actionable insights for interventions. However, limitations include reliance on animal studies, a lack of longitudinal human data, and underrepresentation of diverse global populations. The focus on metabolic outcomes overlooks other areas, like mental health and immune function. Challenges in measuring preconception factors and the exclusion of non-English studies may also limit generalizability and introduce bias.

## 9. Conclusions

This manuscript highlights the critical role of parental preconception health in shaping offspring outcomes through nutrition, body weight, and lifestyle factors. It emphasizes the importance of both maternal and paternal preconception status in determining long-term health trajectories via mechanisms such as epigenetic modifications and metabolic programming. While maternal health has been widely studied, this review underscores the emerging importance of paternal factors, advocating for a holistic approach to preconception care. Practical implications can be included but not limited to interventions targeting improved diet, nutrient sufficiency, weight management, and physical activity during the preconception period that can significantly reduce the risk of chronic diseases across generations. Public health policies that address parental preconception health can play a transformative role in breaking the cycle of intergenerational disease and promoting healthier futures.

## References

[1] Aris I.M., Fleisch A.F., Oken E. (2018). Developmental Origins of Disease: Emerging Prenatal Risk Factors and Future Disease Risk. Curr. Epidemiol. Rep..

[2] Hieronimus B., Ensenauer R. (2021). Influence of Maternal and Paternal Pre-Conception Overweight/Obesity on Offspring Outcomes and Strategies for Prevention. Eur. J. Clin. Nutr..

[3] Sharp G.C., Lawlor D.A. (2019). Paternal Impact on the Life Course Development of Obesity and Type 2 Diabetes in the Offspring. Diabetologia.

[4] Dahlen C.R., Amat S., Caton J.S., Crouse M.S., Da Silva Diniz W.J., Reynolds L.P. (2023). Paternal Effects on Fetal Programming. Anim. Reprod..

[5] Eberle C., Kirchner M.F., Herden R., Stichling S. (2020). Paternal Metabolic and Cardiovascular Programming of Their Offspring: A Systematic Scoping Review. PLoS ONE.

[6] Dimofski P., Meyre D., Dreumont N., Leininger-Muller B. (2021). Consequences of Paternal Nutrition on Offspring Health and Disease. Nutrients.

[7] Fullston T., Teague E.M.C.O., Palmer N.O., Deblasio M.J., Mitchell M., Corbett M., Print C.G., Owens J.A., Lane M. (2013). Paternal Obesity Initiates Metabolic Disturbances in Two Generations of Mice with Incomplete Penetrance to the F2 Generation and Alters the Transcriptional Profile of Testis and Sperm MicroRNA Content. FASEB J..

[8] Raad G., Hazzouri M., Bottini S., Trabucchi M., Azoury J., Grandjean V. (2017). Paternal Obesity: How Bad Is It for Sperm Quality and Progeny Health?. Basic Clin. Androl..

[9] Soubry A., Schildkraut J.M., Murtha A., Wang F., Huang Z., Bernal A., Kurtzberg J., Jirtle R.L., Murphy S.K., Hoyo C. (2013). Paternal Obesity Is Associated with IGF2 Hypomethylation in Newborns: Results from a Newborn Epigenetics Study (NEST) Cohort. BMC Med..

[10] Bromfield J.J., Schjenken J.E., Chin P.Y., Care A.S., Jasper M.J., Robertson S.A. (2014). Maternal Tract Factors Contribute to Paternal Seminal Fluid Impact on Metabolic Phenotype in Offspring. Proc. Natl. Acad. Sci. USA.

[11] Hardy K., Spanos S. (2002). Growth Factor Expression and Function in the Human and Mouse Preimplantation Embryo. J. Endocrinol..

[12] Zheng X., Li Z., Wang G., Wang H., Zhou Y., Zhao X., Cheng C.Y., Qiao Y., Sun F. (2021). Sperm Epigenetic Alterations Contribute to Inter- and Transgenerational Effects of Paternal Exposure to Long-Term Psychological Stress via Evading Offspring Embryonic Reprogramming. Cell Discov..

[13] Xu X., Miao Z., Sun M., Wan B. (2021). Epigenetic Mechanisms of Paternal Stress in Offspring Development and Diseases. Int. J. Genom..

[14] Billah M.M., Khatiwada S., Morris M.J., Maloney C.A. (2022). Effects of Paternal Overnutrition and Interventions on Future Generations. Int. J. Obes..

[15] Ben Maamar M., King S.E., Nilsson E., Beck D., Skinner M.K. (2020). Epigenetic Transgenerational Inheritance of Parent-of-Origin Allelic Transmission of Outcross Pathology and Sperm Epimutations: Epigenetic Transgenerational Parent-of-Origin Allelic Transmission. Dev. Biol..

[16] Mousa A., Naqash A., Lim S. (2019). Macronutrient and Micronutrient Intake during Pregnancy: An Overview of Recent Evidence. Nutrients.

[17] Marshall N.E., Abrams B., Barbour L.A., Catalano P., Christian P., Friedman J.E., Hay W.W., Hernandez T.L., Krebs N.F., Oken E. (2022). The Importance of Nutrition in Pregnancy and Lactation: Lifelong Consequences. Am. J. Obstet. Gynecol..

[18] Lowensohn R.I., Stadler D.D., Naze C. (2016). Current Concepts of Maternal Nutrition. Obstet. Gynecol. Surv..

[19] Li X., Zeng Y.m., Luo Y.d., He J., Luo B.w., Lu X.c., Zhu L.l. (2023). Effects of Folic Acid and Folic Acid plus Zinc Supplements on the Sperm Characteristics and Pregnancy Outcomes of Infertile Men: A Systematic Review and Meta-Analysis. Heliyon.

[20] Crider K.S., Qi Y.P., Yeung L.F., Mai C.T., Head Zauche L., Wang A., Daniels K., Williams J.L. (2022). Folic Acid and the Prevention of Birth Defects: 30 Years of Opportunity and Controversies. Annu. Rev. Nutr..

[21] Levine S.Z., Kodesh A., Viktorin A., Smith L., Uher R., Reichenberg A., Sandin S. (2018). Association of Maternal Use of Folic Acid and Multivitamin Supplements in the Periods before and during Pregnancy with the Risk of Autism Spectrum Disorder in Offspring. JAMA Psychiatry.

[22] Chambers T.J.G., Morgan M.D., Heger A.H., Sharpe R.M., Drake A.J. (2016). High-Fat Diet Disrupts Metabolism in Two Generations of Rats in a Parent-of-Origin Specific Manner. Sci. Rep..

[23] De-Regil L.M., Fernández-Gaxiola A.C., Dowswell T., Peña-Rosas J.P. (2010). Effects and Safety of Periconceptional Folate Supplementation for Preventing Birth Defects. Cochrane Database Syst. Rev..

[24] Crott J.W. (2017). Effects of Altered Parental Folate and One-Carbon Nutrient Status on Offspring Growth and Metabolism. Mol. Aspects Med..

[25] McCoy C.R., Jackson N.L., Brewer R.L., Moughnyeh M.M., Smith D.L., Clinton S.M. (2018). A Paternal Methyl Donor Depleted Diet Leads to Increased Anxiety- and Depression-like Behavior in Adult Rat Offspring. Biosci. Rep..

[26] Lambrot R., Xu C., Saint-Phar S., Chountalos G., Cohen T., Paquet M., Suderman M., Hallett M., Kimmins S. (2013). Low Paternal Dietary Folate Alters the Mouse Sperm Epigenome and Is Associated with Negative Pregnancy Outcomes. Nat. Commun..

[27] Martín-Calvo N., Mínguez-Alarcón L., Gaskins A.J., Nassan F.L., Williams P.L., Souter I., Hauser R., Chavarro J.E. (2019). Paternal Preconception Folate Intake in Relation to Gestational Age at Delivery and Birthweight of Newborns Conceived through Assisted Reproduction. Reprod. Biomed. Online.

[28] Yuan H.F., Zhao K., Zang Y., Liu C.Y., Hu Z.Y., Wei J.J., Zhou T., Li Y., Zhang H.P. (2017). Effect of Folate Deficiency on Promoter Methylation and Gene Expression of Esr1, Cav1, and Elavl1, and Its Influence on Spermatogenesis. Oncotarget.

[29] Swayne B.G., Kawata A., Behan N.A., Williams A., Wade M.G., MacFarlane A.J., Yauk C.L. (2012). Investigating the Effects of Dietary Folic Acid on Sperm Count, DNA Damage and Mutation in Balb/c Mice. Mutat. Res. Fund. Mol. Mech. Mutagen..

[30] Tomizawa H., Matsuzawa D., Ishii D., Matsuda S., Kawai K., Mashimo Y., Sutoh C., Shimizu E. (2015). Methyl-Donor Deficiency in Adolescence Affects Memory and Epigenetic Status in the Mouse Hippocampus. Genes Brain Behav..

[31] Kim H.W., Kim K.N., Choi Y.J., Chang N. (2013). Effects of Paternal Folate Deficiency on the Expression of Insulin-like Growth Factor-2 and Global DNA Methylation in the Fetal Brain. Mol. Nutr. Food Res..

[32] Mejos K.K., Kim H.W., Lim E.M., Chang N. (2013). Effects of Parental Folate Deficiency on the Folate Content, Global DNA Methylation, and Expressions of FRα, IGF-2 and IGF-1R in the Postnatal Rat Liver. Nutr. Res. Pract..

[33] Li X., Duan X., Tan D., Zhang B., Xu A., Qiu N., Chen Z. (2023). Iron Deficiency and Overload in Men and Woman of Reproductive Age, and Pregnant Women. Reprod. Toxicol..

[34] Zimmermann M.B., Boelaert K. (2015). Iodine Deficiency and Thyroid Disorders. Lancet Diabetes Endocrinol..

[35] Pérez-López F.R., Pasupuleti V., Mezones-Holguin E., Benites-Zapata V.A., Thota P., Deshpande A., Hernandez A.V. (2015). Effect of Vitamin D Supplementation during Pregnancy on Maternal and Neonatal Outcomes: A Systematic Review and Meta-Analysis of Randomized Controlled Trials. Fertil. Steril..

[36] Stephenson J., Heslehurst N., Hall J., Schoenaker D.A.J.M., Hutchinson J., Cade J.E., Poston L., Barrett G., Crozier S.R., Barker M. (2018). Before the Beginning: Nutrition and Lifestyle in the Preconception Period and Its Importance for Future Health. Lancet.

[37] Watkins A.J., Sinclair K.D. (2014). Paternal Low Protein Diet Affects Adult Offspring Cardiovascular and Metabolic Function in Mice. Am. J. Physiol. Heart Circ. Physiol..

[38] Murray C.J.L., Barber R.M., Foreman K.J., Ozgoren A.A., Abd-Allah F., Abera S.F., Aboyans V., Abraham J.P., Abubakar I., Abu-Raddad L.J. (2015). Global, Regional, and National Disability-Adjusted Life Years (DALYs) for 306 Diseases and Injuries and Healthy Life Expectancy (HALE) for 188 Countries, 1990-2013: Quantifying the Epidemiological Transition. Lancet.

[39] Wu G., Imhoff-Kunsch B., Girard A.W. (2012). Biological Mechanisms for Nutritional Regulation of Maternal Health and Fetal Development. Paediatr. Perinat. Epidemiol..

[40] Cetin I., Mandò C., Calabrese S. (2013). Maternal Predictors of Intrauterine Growth Restriction. Curr. Opin. Clin. Nutr. Metab. Care.

[41] Dean S.V., Lassi Z.S., Imam A.M., Bhutta Z.A. (2014). Preconception Care: Nutritional Risks and Interventions. Reprod. Health.

[42] Przybysz P., Kruszewski A., Kacperczyk-Bartnik J., Romejko-Wolniewicz E. (2023). The Impact of Maternal Plant-Based Diet on Obstetric and Neonatal Outcomes—A Cross-Sectional Study. Nutrients.

[43] Ouyang F., Wang X., Wells J.C., Wang X., Shen L., Zhang J. (2023). Maternal Pre-Pregnancy Nutritional Status and Infant Birth Weight in Relation to 0–2 Year-Growth Trajectory and Adiposity in Term Chinese Newborns with Appropriate Birth Weight-for-Gestational Age. Nutrients.

[44] Terada S., Isumi A., Doi S., Fujiwara T. (2024). Association of Maternal Pre-Pregnancy Body Mass Index with Resilience and Prosociality of the Offspring Aged 6–7 Years Old: A Population-Based Cohort Study in Japan. Eur. Child. Adolesc. Psychiatry.

[45] Nguyen P.H., Young M.F., Khuong L.Q., Tran L.M., Duong T.H., Nguyen H.C., Martorell R., Ramakrishnan U. (2021). Maternal Preconception Body Size and Early Childhood Growth during Prenatal and Postnatal Periods Are Positively Associated with Child-Attained Body Size at Age 6-7 Years: Results from a Follow-up of the PRECONCEPT Trial. J. Nutr..

[46] Soliman A., De Sanctis V., Alaaraj N., Ahmed S., Alyafei F., Hamed N., Soliman N. (2021). Early and Long-Term Consequences of Nutritional Stunting: From Childhood to Adulthood. Acta Biomed..

[47] Black R.E., Victora C.G., Walker S.P., Bhutta Z.A., Christian P., De Onis M., Ezzati M., Grantham-Mcgregor S., Katz J., Martorell R. (2013). Maternal and Child Undernutrition and Overweight in Low-Income and Middle-Income Countries. Lancet.

[48] Knop M.R., Nagashima-Hayashi M., Lin R., Saing C.H., Ung M., Oy S., Yam E.L.Y., Zahari M., Yi S. (2024). Impact of MHealth Interventions on Maternal, Newborn, and Child Health from Conception to 24 Months Postpartum in Low- and Middle-Income Countries: A Systematic Review. BMC Med..

[49] Han Z., Mulla S., Beyene J., Liao G., McDonald S.D. (2011). Maternal Underweight and the Risk of Preterm Birth and Low Birth Weight: A Systematic Review and Meta-Analyses. Int. J. Epidemiol..

[50] Nogues P., Dos Santos E., Couturier-Tarrade A., Berveiller P., Arnould L., Lamy E., Grassin-Delyle S., Vialard F., Dieudonne M.N. (2021). Maternal Obesity Influences Placental Nutrient Transport, Inflammatory Status, and Morphology in Human Term Placenta. J. Clin. Endocrinol. Metab..

[51] Perumal N., Bassani D.G., Roth D.E. (2018). Use and Misuse of Stunting as a Measure of Child Health. J. Nutr..

[52] Odhiambo J.F., Pankey C.L., Ghnenis A.B., Ford S.P. (2020). A Review of Maternal Nutrition during Pregnancy and Impact on the Offspring through Development: Evidence from Animal Models of over-and Undernutrition. Int. J. Environ. Res. Public Health.

[53] Reynolds C.M., Vickers M.H. (2022). Editorial: Maternal Diet and Offspring Health. Front. Nutr..

[54] Lewis A.J., Austin E., Knapp R., Vaiano T., Galbally M. (2015). Perinatal Maternal Mental Health, Fetal Programming and Child Development. Healthcare.

[55] Lillycrop K.A., Burdge G.C. (2011). Epigenetic Changes in Early Life and Future Risk of Obesity. Int. J. Obes..

[56] Matzkin L.M., Johnson S., Paight C., Markow T.A. (2013). Preadult Parental Diet Affects Offspring Development and Metabolism in Drosophila Melanogaster. PLoS ONE.

[57] Entringer S., Buss C., Wadhwa P.D. (2015). Prenatal Stress, Development, Health and Disease Risk: A Psychobiological Perspective-2015 Curt Richter Award Paper. Psychoneuroendocrinology.

[58] Fullston T., Palmer N.O., Owens J.A., Mitchell M., Bakos H.W., Lane M. (2012). Diet-Induced Paternal Obesity in the Absence of Diabetes Diminishes the Reproductive Health of Two Subsequent Generations of Mice. Hum. Reprod..

[59] Salto R., Girón M.D., Manzano M., Martín M.J., Vílchez J.D., Bueno-Vargas P., Cabrera E., Pérez-Alegre M., Andujar E., Rueda R. (2020). Programming Skeletal Muscle Metabolic Flexibility in Offspring of Male Rats in Response to Maternal Consumption of Slow Digesting Carbohydrates during Pregnancy. Nutrients.

[60] Morgan H.L., Aljumah A., Rouillon C., Watkins A.J. (2021). Paternal Low Protein Diet and the Supplementation of Methyl-Donors Impact Fetal Growth and Placental Development in Mice. Placenta.

[61] Furse S., Morgan H.L., Koulman A., Watkins A.J. (2023). Characterisation of the Paternal Influence on Intergenerational Offspring Cardiac and Brain Lipid Homeostasis in Mice. Int. J. Mol. Sci..

[62] Cao R., Xie J., Zhang L. (2022). Abnormal Methylation Caused by Folic Acid Deficiency in Neural Tube Defects. Open Life Sci..

[63] Shi Q., Qi K. (2023). Developmental Origins of Health and Disease: Impact of Paternal Nutrition and Lifestyle. Pediatr. Investig..

[64] Al Aboud N.M., Jialal I. (2018). Tupper Connor Genetics, Epigenetic Mechanism [Updated 14 August 2023].

[65] Mierziak J., Kostyn K., Boba A., Czemplik M., Kulma A., Wojtasik W. (2021). Influence of the Bioactive Diet Components on the Gene Expression Regulation. Nutrients.

[66] Banik A., Kandilya D., Ramya S., Stünkel W., Chong Y.S., Thameem Dheen S. (2017). Maternal Factors That Induce Epigenetic Changes Contribute to Neurological Disorders in Offspring. Genes.

[67] da Cruz R.S., Carney E.J., Clarke J., Cao H., Cruz M.I., Benitez C., Jin L., Fu Y., Cheng Z., Wang Y. (2018). Paternal Malnutrition Programs Breast Cancer Risk and Tumor Metabolism in Offspring. Breast Cancer Res..

[68] Sabet J.A., Park L.K., Iyer L.K., Tai A.K., Koh G.Y., Pfalzer A.C., Parnell L.D., Mason J.B., Liu Z., Byun A.J. (2016). Paternal B Vitamin Intake Is a Determinant of Growth, Hepatic Lipid Metabolism and Intestinal Tumor Volume in Female Apc1638N Mouse Offspring. PLoS ONE.

[69] Kanwal R., Gupta S. (2012). Epigenetic Modifications in Cancer. Clin. Genet..

[70] Wu T., Cai W., Chen X. (2023). Epigenetic Regulation of Neurotransmitter Signaling in Neurological Disorders. Neurobiol. Dis..

[71] Pullar J., Wickramasinghe K., Demaio A.R., Roberts N., Perez-Blanco1 K.M., Noonan K., Townsend N. (2019). The Impact of Maternal Nutrition on Offspring’s Risk of Non-Communicable Diseases in Adulthood: A Systematic Review. J. Glob. Health.

[72] Day J., Savani S., Krempley B.D., Nguyen M., Kitlinska J.B. (2016). Influence of Paternal Preconception Exposures on Their Offspring: Through Epigenetics to Phenotype. Am. J. Stem Cells.

[73] Fuemmeler B.F., Lovelady C.A., Zucker N.L., Østbye T. (2013). Parental Obesity Moderates the Relationship between Childhood Appetitive Traits and Weight. Obesity.

[74] Heslehurst N., Vieira R., Akhter Z., Bailey H., Slack E., Ngongalah L., Pemu A., Rankin J. (2019). The Association between Maternal Body Mass Index and Child Obesity: A Systematic Review and Meta-Analysis. PLoS Med..

[75] Den Harink T., Roelofs M.J.M., Limpens J., Painter R.C., Roseboom T.J., Van Deutekom A.W. (2022). Maternal Obesity in Pregnancy and Children’s Cardiac Function and Structure: A Systematic Review and Meta-Analysis of Evidence from Human Studies. PLoS ONE.

[76] Edlow A.G. (2017). Maternal Obesity and Neurodevelopmental and Psychiatric Disorders in Offspring. Prenat. Diagn..

[77] Adane A.A., Mishra G.D., Tooth L.R. (2016). Maternal Pre-Pregnancy Obesity and Childhood Physical and Cognitive Development of Children: A Systematic Review. Int. J. Obes..

[78] Álvarez-Bueno C., Cavero-Redondo I., Lucas-de la Cruz L., Notario-Pacheco B., Martínez-Vizcaíno V. (2017). Association between Pre-Pregnancy Overweight and Obesity and Children’s Neurocognitive Development: A Systematic Review and Meta-Analysis of Observational Studies. Int. J. Epidemiol..

[79] Lei X.Y., Li Y.J., Ou J.J., Li Y.M. (2019). Association between Parental Body Mass Index and Autism Spectrum Disorder: A Systematic Review and Meta-Analysis. Eur. Child. Adolesc. Psychiatry.

[80] Li L., Lagerberg T., Chang Z., Cortese S., Rosenqvist M.A., Almqvist C., D’Onofrio B.M., Hegvik T.A., Hartman C., Chen Q. (2021). Maternal Pre-Pregnancy Overweight/Obesity and the Risk of Attention-Deficit/Hyperactivity Disorder in Offspring: A Systematic Review, Metaanalysis and Quasi-Experimental Family-Based Study. Int. J. Epidemiol..

[81] Li Y.M., Ou J.J., Liu L., Zhang D., Zhao J.P., Tang S.Y. (2016). Association Between Maternal Obesity and Autism Spectrum Disorder in Offspring: A Meta-Analysis. J. Autism. Dev. Disord..

[82] Sanchez C.E., Barry C., Sabhlok A., Russell K., Majors A., Kollins S.H., Fuemmeler B.F. (2018). Maternal Pre-Pregnancy Obesity and Child Neurodevelopmental Outcomes: A Meta-Analysis. Obesity Rev..

[83] Yeung A.Y., Tadi P. (2024). Physiology, Obesity Neurohormonal Appetite And Satiety Control. StatPearls [Internet].

[84] Zheng L., Yang L., Guo Z., Yao N., Zhang S., Pu P. (2023). Obesity and Its Impact on Female Reproductive Health: Unraveling the Connections. Front. Endocrinol..

[85] Santangelo C., Varì R., Scazzocchio B., Filesi C., Masella R. (2014). Management of Reproduction and Pregnancy Complications in Maternal Obesity: Which Role for Dietary Polyphenols?. BioFactors.

[86] Tahiri I., Llana S.R., Fos-Domènech J., Milà-Guash M., Toledo M., Haddad-Tóvolli R., Claret M., Obri A. (2024). Paternal Obesity Induces Changes in Sperm Chromatin Accessibility and Has a Mild Effect on Offspring Metabolic Health. Heliyon.

[87] de Castro Barbosa T., Ingerslev L.R., Alm P.S., Versteyhe S., Massart J., Rasmussen M., Donkin I., Sjögren R., Mudry J.M., Vetterli L. (2016). High-Fat Diet Reprograms the Epigenome of Rat Spermatozoa and Transgenerationally Affects Metabolism of the Offspring. Mol. Metab..

[88] Ng S.F., Lin R.C.Y., Laybutt D.R., Barres R., Owens J.A., Morris M.J. (2010). Chronic High-Fat Diet in Fathers Programs β 2-Cell Dysfunction in Female Rat Offspring. Nature.

[89] Öst A., Lempradl A., Casas E., Weigert M., Tiko T., Deniz M., Pantano L., Boenisch U., Itskov P.M., Stoeckius M. (2014). Paternal Diet Defines Offspring Chromatin State and Intergenerational Obesity. Cell.

[90] Raad G., Serra F., Martin L., Derieppe M.A., Gilleron J., Costa V.L., Pisani D.F., Amri E.Z., Trabucchi M., Grandjean V. (2021). Paternal Multigenerational Exposure to an Obesogenic Diet Drives Epigenetic Predisposition to Metabolic Diseases in Mice. eLife.

[91] Aizawa S., Tochihara A., Yamamuro Y. (2022). Paternal High-Fat Diet Alters Triglyceride Metabolism-Related Gene Expression in Liver and White Adipose Tissue of Male Mouse Offspring. Biochem. Biophys. Rep..

[92] Fullston T., McPherson N.O., Owens J.A., Kang W.X., Sandeman L.Y., Lane M. (2015). Paternal Obesity Induces Metabolic and Sperm Disturbances in Male Offspring That Are Exacerbated by Their Exposure to an “Obesogenic” Diet. Physiol. Rep..

[93] Masuyama H., Mitsui T., Eguchi T., Tamada S., Hiramatsu Y. (2016). The Effects of Paternal High-Fat Diet Exposure on Offspring Metabolism with Epigenetic Changes in the Mouse Adiponectin and Leptin Gene Promoters. Am. J. Physiol. Endocrinol. Metab..

[94] Casasnovas J., Damron C.L., Jarrell J., Orr K.S., Bone R.N., Archer-Hartmann S., Azadi P., Kua K.L. (2021). Offspring of Obese Dams Exhibit Sex-Differences in Pancreatic Heparan Sulfate Glycosaminoglycans and Islet Insulin Secretion. Front. Endocrinol..

[95] Ramaraju G.A., Teppala S., Prathigudupu K., Kalagara M., Thota S., Kota M., Cheemakurthi R. (2018). Association between Obesity and Sperm Quality. Andrologia.

[96] Panera N., Mandato C., Crudele A., Bertrando S., Vajro P., Alisi A. (2022). Genetics, Epigenetics and Transgenerational Transmission of Obesity in Children. Front. Endocrinol..

[97] Cabler S., Agarwal A., Flint M., Du Plessis S.S. (2010). Obesity: Modern Man’s Fertility Nemesis. Asian J. Androl..

[98] Kort H.I., Massey J.B., Elsner C.W., Mitchell-Leef D., Shapiro D.B., Witt M.A., Roudebush W.E. (2006). Impact of Body Mass Index Values on Sperm Quantity and Quality. J. Androl..

[99] Macdonald A.A., Stewart A.W., Farquhar C.M. (2013). Body Mass Index in Relation to Semen Quality and Reproductive Hormones in New Zealand Men: A Cross-Sectional Study in Fertility Clinics. Hum. Reprod..

[100] Hammoud A.O., Wilde N., Gibson M., Parks A., Carrell D.T., Meikle A.W. (2008). Male Obesity and Alteration in Sperm Parameters. Fertil. Steril..

[101] González-Domínguez Á., Jurado-Sumariva L., Domínguez-Riscart J., Saez-Benito A., González-Domínguez R. (2024). Parental Obesity Predisposes to Exacerbated Metabolic and Inflammatory Disturbances in Childhood Obesity within the Framework of an Altered Profile of Trace Elements. Nutr. Diabetes.

[102] Rodgers A.B., Morgan C.P., Leu N.A., Bale T.L. (2015). Transgenerational Epigenetic Programming via Sperm MicroRNA Recapitulates Effects of Paternal Stress. Proc. Natl. Acad. Sci. USA.

[103] Ly L., Chan D., Trasler J.M. (2015). Developmental Windows of Susceptibility for Epigenetic Inheritance through the Male Germline. Semin. Cell Dev. Biol..

[104] Almujaydil M.S. (2023). The Role of Dietary Nutrients in Male Infertility: A Review. Life.

[105] Jiao P., Lu H., Hao L., Degen A.A., Cheng J., Yin Z., Mao S., Xue Y. (2024). Nutrigenetic and Epigenetic Mechanisms of Maternal Nutrition–Induced Glucolipid Metabolism Changes in the Offspring. Nutr. Rev..

[106] Gilley S.P., Harrall K.K., Friedman C., Glueck D.H., Cohen C.C., Perng W., Sauder K.A., Krebs N.F., Shankar K., Dabelea D. (2023). Association of Maternal BMI and Rapid Infant Weight Gain with Childhood Body Size and Composition. Pediatrics.

[107] Sasidharan Pillai S., Gagnon C.A., Foster C., Ashraf A.P. (2024). Exploring the Gut Microbiota: Key Insights Into Its Role in Obesity, Metabolic Syndrome, and Type 2 Diabetes. J. Clin. Endocrinol. Metab..

[108] Li R.L., Kang S. (2024). Rewriting Cellular Fate: Epigenetic Interventions in Obesity and Cellular Programming. Mol. Med..

[109] Champroux A., Cocquet J., Henry-Berger J., Drevet J.R., Kocer A. (2018). A Decade of Exploring the Mammalian Sperm Epigenome: Paternal Epigenetic and Transgenerational Inheritance. Front. Cell Dev. Biol..

[110] Harmancıoğlu B., Kabaran S. (2023). Maternal High Fat Diets: Impacts on Offspring Obesity and Epigenetic Hypothalamic Programming. Front. Genet..

[111] Braun K., Champagne F.A. (2014). Paternal Influences on Offspring Development: Behavioural and Epigenetic Pathways. J. Neuroendocrinol..

[112] Sanchez-Garrido M.A., Ruiz-Pino F., Velasco I., Barroso A., Fernandois D., Heras V., Manfredi-Lozano M., Vazquez M.J., Castellano J.M., Roa J. (2018). Intergenerational Influence of Paternal Obesity on Metabolic and Reproductive Health Parameters of the Offspring: Male-Preferential Impact and Involvement of Kiss1-Mediated Pathways. Endocrinology.

[113] McPherson N.O., Owens J.A., Fullston T., Lane M. (2015). Preconception Diet or Exercise Intervention in Obese Fathers Normalizes Sperm MicroRNA Profile and Metabolic Syndrome in Female Offspring. Am. J. Physiol. Endocrinol. Metab..

[114] McIntyre H.D., Catalano P., Zhang C., Desoye G., Mathiesen E.R., Damm P. (2019). Gestational Diabetes Mellitus. Nat. Rev. Dis. Primers.

[115] Yessoufou A., Moutairou K. (2011). Maternal Diabetes in Pregnancy: Early and Long-Term Outcomes on the Offspring and the Concept of “Metabolic Memory”. Exp. Diabetes Res..

[116] Aiken C.E., Ozanne S.E. (2014). Transgenerational Developmental Programming. Hum. Reprod. Update.

[117] Nteeba J., Nteeba J., Varberg K.M., Varberg K.M., Scott R.L., Scott R.L., Simon M.E., Simon M.E., Iqbal K., Iqbal K. (2020). Poorly Controlled Diabetes Mellitus Alters Placental Structure, Efficiency, and Plasticity. BMJ Open Diabetes Res. Care.

[118] Dabelea D., Crume T. (2011). Maternal Environment and the Transgenerational Cycle of Obesity and Diabetes. Diabetes.

[119] Xiang A.H., Wang X., Martinez M.P., Walthall J.C., Curry E.S., Page K., Buchanan T.A., Coleman K.J., Getahun D. (2015). Association of Maternal Diabetes with Autism in Offspring. JAMA.

[120] Linares Segovia B., Gutiérrez Tinoco M., Izquierdo Arrizon A., Guízar Mendoza J.M., Amador Licona N. (2012). Long-Term Consequences for Offspring of Paternal Diabetes and Metabolic Syndrome. Exp. Diabetes Res..

[121] Gilbert E.R., Liu D. (2012). Epigenetics the Missing Link to Understanding β-Cell Dysfunction in the Pathogenesis of Type 2 Diabetes. Epigenetics.

[122] Zhu Z., Cao F., Li X. (2019). Epigenetic Programming and Fetal Metabolic Programming. Front. Endocrinol..

[123] Penesova A., Bunt J.C., Bogardus C., Krakoff J. (2010). Effect of Paternal Diabetes on Pre-Diabetic Phenotypes in Adult Offspring. Diabetes Care.

[124] Wei Y., Yang C.R., Wei Y.P., Zhao Z.A., Hou Y., Schatten H., Sun Q.Y. (2014). Paternally Induced Transgenerational Inheritance of Susceptibility to Diabetes in Mammals. Proc. Natl. Acad. Sci. USA.

[125] Wannamethee S.G., Lawlor D.A., Whincup P.H., Walker M., Ebrahim S., Davey-Smith G. (2004). Birthweight of Offspring and Paternal Insulin Resistance and Paternal Diabetes in Late Adulthood: Cross Sectional Survey. Diabetologia.

[126] Lindsay R.S., Dabelea D., Roumain J., Hanson R.L., Bennett P.H., Knowler W.C. (2000). Type 2 and Low Birth Weight: The Role of Paternal Inheritance in the Association of Low Birth Weight and Diabetes. Diabetes.

[127] Dornhorst A., Rossi M. (1998). Risk and Prevention of Type 2 Diabetes in Women with Gestational Diabetes. Diabetes Care.

[128] Kusuyama J., Alves-Wagner A.B., Makarewicz N.S., Goodyear L.J. (2020). Effects of Maternal and Paternal Exercise on Offspring Metabolism. Nat. Metab..

[129] Falcão-Tebas F., Kuang J., Arceri C., Kerris J.P., Andrikopoulos S., Marin E.C., McConell G.K. (2019). Four Weeks of Exercise Early in Life Reprograms Adult Skeletal Muscle Insulin Resistance Caused by a Paternal High-Fat Diet. J. Physiol..

[130] Mega F., de Meireles A.L.F., Piazza F.V., Spindler C., Segabinazi E., dos Santos Salvalaggio G., Achaval M., Marcuzzo S. (2018). Paternal Physical Exercise Demethylates the Hippocampal DNA of Male Pups without Modifying the Cognitive and Physical Development. Behav. Brain Res..

[131] Vieira de Sousa Neto I., Fontes W., Prestes J., de Cassia Marqueti R. (2021). Impact of Paternal Exercise on Physiological Systems in the Offspring. Acta Physiol..

[132] Denham J., O’Brien B.J., Harvey J.T., Charchar F.J. (2015). Genome-Wide Sperm DNA Methylation Changes after 3 Months of Exercise Training in Humans. Epigenomics.

[133] Murashov A.K., Pak E.S., Koury M., Ajmera A., Jeyakumar M., Parker M., Williams O., Ding J., Walters D., Neufer P.D. (2016). Paternal Long-Term Exercise Programs Offspring for Low Energy Expenditure and Increased Risk for Obesity in Mice. FASEB J..

[134] Barrès R., Zierath J.R. (2016). The Role of Diet and Exercise in the Transgenerational Epigenetic Landscape of T2DM. Nat. Rev. Endocrinol..

[135] Zheng J., Xiao X., Zhang Q., Yu M., Xu J., Wang Z. (2014). Maternal High-Fat Diet Modulates Hepatic Glucose, Lipid Homeostasis and Gene Expression in the PPAR Pathway in the Early Life of Offspring. Int. J. Mol. Sci..

[136] Stanford K.I., Lee M.Y., Getchell K.M., So K., Hirshman M.F., Goodyear L.J. (2015). Exercise before and during Pregnancy Prevents the Deleterious Effects of Maternal High-Fat Feeding on Metabolic Health of Male Offspring. Diabetes.

[137] Claycombe-Larson K.G., Bundy A.N., Roemmich J.N. (2020). Paternal High-Fat Diet and Exercise Regulate Sperm MiRNA and Histone Methylation to Modify Placental Inflammation, Nutrient Transporter MRNA Expression and Fetal Weight in a Sex-Dependent Manner. J. Nutr. Biochem..

[138] Stanford K.I., Rasmussen M., Baer L.A., Lehnig A.C., Rowland L.A., White J.D., So K., De Sousa-Coelho A.L., Hirshman M.F., Patti M.E. (2018). Paternal Exercise Improves Glucose Metabolism in Adult Offspring. Diabetes.

[139] Costa-Júnior J.M., Ferreira S.M., Kurauti M.A., Bernstein D.L., Ruano E.G., Kameswaran V., Schug J., Freitas-Dias R., Zoppi C.C., Boschero A.C. (2022). Paternal Exercise Improves the Metabolic Health of Offspring via Epigenetic Modulation of the Germline. Int. J. Mol. Sci..

[140] Spindler C., Segabinazi E., De Meireles A.L.F., Piazza F.V., Mega F., Dos Santos Salvalaggio G., Achaval M., Elsner V.R., Marcuzzo S. (2019). Paternal Physical Exercise Modulates Global DNA Methylation Status in the Hippocampus of Male Rat Offspring. Neural Regen. Res..

[141] Krout D., Roemmich J.N., Bundy A., Garcia R.A., Yan L., Claycombe-Larson K.J. (2018). Paternal Exercise Protects Mouse Offspring from High-Fat-Diet-Induced Type 2 Diabetes Risk by Increasing Skeletal Muscle Insulin Signaling. J. Nutr. Biochem..

[142] Kankowski L., Ardissino M., McCracken C., Lewandowski A.J., Leeson P., Neubauer S., Harvey N.C., Petersen S.E., Raisi-Estabragh Z. (2022). The Impact of Maternal Obesity on Offspring Cardiovascular Health: A Systematic Literature Review. Front. Endocrinol..

[143] Şanlı E., Kabaran S. (2019). Maternal Obesity, Maternal Overnutrition and Fetal Programming: Effects of Epigenetic Mechanisms on the Development of Metabolic Disorders. Curr. Genom..

[144] Radford B.N., Han V.K.M. (2019). Offspring from Maternal Nutrient Restriction in Mice Show Variations in Adult Glucose Metabolism Similar to Human Fetal Growth Restriction. J. Dev. Orig. Health Dis..

[145] Cooke C.L., Zhao L., Gysler S., Arany E., Regnault T.R.H. (2014). Sex-Specific Effects of Low Protein Diet on in Utero Programming of Renal G-Protein Coupled Receptors. J. Dev. Orig. Health Dis..

[146] Rando O.J., Simmons R.A. (2015). I’m Eating for Two: Parental Dietary Effects on Offspring Metabolism. Cell.

[147] McKay J.A., Mathers J.C. (2016). Maternal Folate Deficiency and Metabolic Dysfunction in Offspring. Proc. Nutr. Soc..

[148] Watkins A.J., Dias I., Tsuro H., Allen D., Emes R.D., Moreton J., Wilson R., Ingram R.J.M., Sinclair K.D. (2018). Paternal Diet Programs Offspring Health through Sperm- and Seminal Plasma-Specific Pathways in Mice. Proc. Natl. Acad. Sci. USA.

[149] McPherson N.O., Fullston T., Kang W.X., Sandeman L.Y., Corbett M.A., Owens J.A., Lane M. (2016). Paternal Under-Nutrition Programs Metabolic Syndrome in Offspring Which Can Be Reversed by Antioxidant/Vitamin Food Fortification in Fathers. Sci. Rep..

[150] Fante T., Simino L.A.P., Fontana M.F., Reginato A., Ramalheira T.G., Rodrigues H.G., Lisboa P.C., De Moura E.G., Ignácio-Souza L.M., Milanski M. (2022). Maternal High-Fat Diet Consumption Programs Male Offspring to Mitigate Complications in Liver Regeneration. J. Dev. Orig. Health Dis..

[151] Alfaradhi M.Z., Ozanne S.E. (2011). Developmental Programming in Response to Maternal Overnutrition. Front Genet.

[152] Robertson R.C., Kaliannan K., Strain C.R., Ross R.P., Stanton C., Kang J.X. (2018). Maternal Omega-3 Fatty Acids Regulate Offspring Obesity through Persistent Modulation of Gut Microbiota. Microbiome.

[153] Kim S.M., Oh S., Lee S.S., Park S., Hur Y.M., Ansari A.Z., Lee G., Paik M.J., You Y.A., Kim Y.J. (2024). Maternal Diet during Pregnancy Alters the Metabolites in Relation to Metabolic and Neurodegenerative Diseases in Young Adult Offspring. Int. J. Mol. Sci..

[154] Sohi G., Marchand K., Revesz A., Arany E., Hardy D.B. (2011). Maternal Protein Restriction Elevates Cholesterol in Adult Rat Offspring Due to Repressive Changes in Histone Modifications at the Cholesterol 7α-Hydroxylase Promoter. Mol. Endocrinol..

[155] Benatti R.O., Melo A.M., Borges F.O., Ignacio-Souza L.M., Simino L.A.P., Milanski M., Velloso L.A., Torsoni M.A., Torsoni A.S. (2014). Maternal High-Fat Diet Consumption Modulates Hepatic Lipid Metabolism and MicroRNA-122 (MiR-122) and MicroRNA-370 (MiR-370) Expression in Offspring. Br. J. Nutr..

[156] Nelson B.N., Friedman J.E. (2024). Developmental Programming of the Fetal Immune System by Maternal Western-Style Diet: Mechanisms and Implications for Disease Pathways in the Offspring. Int. J. Mol. Sci..

[157] Knuesel I., Chicha L., Britschgi M., Schobel S.A., Bodmer M., Hellings J.A., Toovey S., Prinssen E.P. (2014). Maternal Immune Activation and Abnormal Brain Development across CNS Disorders. Nat. Rev. Neurol..

[158] Boulanger-Bertolus J., Pancaro C., Mashour G.A. (2018). Increasing Role of Maternal Immune Activation in Neurodevelopmental Disorders. Front. Behav. Neurosci..

[159] Sakurada K., Noda Y. (2020). Neurodevelopmental Disorders Induced by Maternal Immune Activation: Toward a Prevention Strategy in the Era of the COVID-19 Pandemic. Psychiatry Int..

[160] Rees G., Brough L., Orsatti G.M., Lodge A., Walker S. (2022). Do Micronutrient and Omega-3 Fatty Acid Supplements Affect Human Maternal Immunity during Pregnancy? A Scoping Review. Nutrients.

[161] McNamara K.B., Van Lieshout E., Simmons L.W. (2014). The Effect of Maternal and Paternal Immune Challenge on Offspring Immunity and Reproduction in a Cricket. J. Evol. Biol..

[162] Eggert H., Kurtz J., Diddens-de Buhr M.F. (2014). Different Effects of Paternal Transgenerational Immune Priming on Survival and Immunity in Step and Genetic Offspring. Proc. R. Soc. B Biol. Sci..

[163] Broadney M.M., Chahal N., Michels K.A., McLain A.C., Ghassabian A., Lawrence D.A., Yeung E.H. (2017). Impact of Parental Obesity on Neonatal Markers of Inflammation and Immune Response. Int. J. Obes..

[164] Ornellas F., Souza-Mello V., Mandarim-de-Lacerda C.A., Aguila M.B. (2016). Combined Parental Obesity Augments Single-Parent Obesity Effects on Hypothalamus Inflammation, Leptin Signaling (JAK/STAT), Hyperphagia, and Obesity in the Adult Mice Offspring. Physiol. Behav..

[165] Bomans K., Schenz J., Tamulyte S., Schaack D., Weigand M.A., Uhle F. (2018). Paternal Sepsis Induces Alterations of the Sperm Methylome and Dampens Offspring Immune Responses-an Animal Study. Clin. Epigenetics.

[166] Cortés-Albornoz M.C., García-Guáqueta D.P., Velez-Van-meerbeke A., Talero-Gutiérrez C. (2021). Maternal Nutrition and Neurodevelopment: A Scoping Review. Nutrients.

[167] Li M., Francis E., Hinkle S.N., Ajjarapu A.S., Zhang C. (2019). Preconception and Prenatal Nutrition and Neurodevelopmental Disorders: A Systematic Review and Meta-Analysis. Nutrients.

[168] Hamner H.C., Nelson J.M., Sharma A.J., Jefferds M.E.D., Dooyema C., Flores-Ayala R., Bremer A.A., Vargas A.J., Casavale K.O., de Jesus J.M. (2022). Improving Nutrition in the First 1000 Days in the United States: A Federal Perspective. Am. J. Public Health.

[169] Berti C., Biesalski H.K., Gärtner R., Lapillonne A., Pietrzik K., Poston L., Redman C., Koletzko B., Cetin I. (2011). Micronutrients in Pregnancy: Current Knowledge and Unresolved Questions. Clin. Nutr..

[170] Guéant J.L., Namour F., Guéant-Rodriguez R.M., Daval J.L. (2013). Folate and Fetal Programming: A Play in Epigenomics?. Trends Endocrinol. Metab..

[171] Chu D., Li L., Jiang Y., Tan J., Ji J., Zhang Y., Jin N., Liu F. (2019). Excess Folic Acid Supplementation before and during Pregnancy and Lactation Activates Fos Gene Expression and Alters Behaviors in Male Mouse Offspring. Front. Neurosci..

[172] Yang X., Sun W., Wu Q., Lin H., Lu Z., Shen X., Chen Y., Zhou Y., Huang L., Wu F. (2022). Excess Folic Acid Supplementation before and during Pregnancy and Lactation Alters Behaviors and Brain Gene Expression in Female Mouse Offspring. Nutrients.

[173] Winther G., Elfving B., Müller H.K., Lund S., Wegener G. (2018). Maternal High-Fat Diet Programs Offspring Emotional Behavior in Adulthood. Neuroscience.

[174] Moser V.C., McDaniel K.L., Woolard E.A., Phillips P.M., Franklin J.N., Gordon C.J. (2017). Impacts of Maternal Diet and Exercise on Offspring Behavior and Body Weights. Neurotoxicol. Teratol..

[175] Jacques A., Chaaya N., Beecher K., Ali S.A., Belmer A., Bartlett S. (2019). The Impact of Sugar Consumption on Stress Driven, Emotional and Addictive Behaviors. Neurosci. Biobehav. Rev..

[176] Sullivan E.L., Nousen E.K., Chamlou K.A. (2014). Maternal High Fat Diet Consumption during the Perinatal Period Programs Offspring Behavior. Physiol. Behav..

[177] Urbonaite G., Knyzeliene A., Bunn F.S., Smalskys A., Neniskyte U. (2022). The Impact of Maternal High-Fat Diet on Offspring Neurodevelopment. Front. Neurosci..

[178] Mort E.J., Heritage S., Jones S., Fowden A.L., Camm E.J. (2023). Sex-Specific Effects of a Maternal Obesogenic Diet High in Fat and Sugar on Offspring Adiposity, Growth, and Behavior. Nutrients.

[179] Pina-Camacho L., Jensen S.K., Gaysina D., Barker E.D. (2015). Maternal Depression Symptoms, Unhealthy Diet and Child Emotional-Behavioural Dysregulation. Psychol. Med..

[180] Cendra-Duarte E., Canals J., Becerra-Tomás N., Jardí C., Martín-Luján F., Arija V. (2024). Maternal Dietary Patterns and Offspring Behavioral Problems. Pediatr. Res..

[181] Gale C.R., Robinson S.M., Godfrey K.M., Law C.M., Schlotz W., O’Callaghan F.J. (2008). Oily Fish Intake during Pregnancy—Association with Lower Hyperactivity but Not with Higher Full-Scale IQ in Offspring. J. Child. Psychol. Psychiatry.

[182] Hibbeln J.R., Davis J.M., Steer C., Emmett P., Rogers I., Williams C., Golding J. (2007). Maternal Seafood Consumption in Pregnancy and Neurodevelopmental Outcomes in Childhood (ALSPAC Study): An Observational Cohort Study. Lancet.

[183] Queiroz M.P., da Silva Lima M., de Melo M.F.F.T., de Menezes Santos Bertozzo C.C., de Araújo D.F., Guerra G.C.B., de Cassia Ramos do Egypto Queiroga R., Soares J.K.B. (2019). Maternal Suppplementation with Conjugated Linoleic Acid Reduce Anxiety and Lipid Peroxidation in the Offspring Brain. J. Affect. Disord..

[184] Jackson C., Barrett D., Shumake J., Gonzalez-Lima F., Lane M. (2014). Maternal Omega-3 Fatty Acid Consumption Decreases Offspring Reactivity to the Stress of a Novel Environment (684.3). FASEB J..

[185] Jackson C., Alhado M., Gonzales E., Shumake J., Barrett D., Gonzalez-Lima F., Lane M.A. (2016). Maternal Consumption of a Diet Lacking Omega-3 Fatty Acids during Development Alters Pup Behavior and Brain Metabolism Later in Life. FASEB J..

[186] Sakayori N., Kikkawa T., Tokuda H., Kiryu E., Yoshizaki K., Kawashima H., Yamada T., Arai H., Kang J.X., Katagiri H. (2016). Maternal Dietary Imbalance between Omega-6 and Omega-3 Polyunsaturated Fatty Acids Impairs Neocortical Development via Epoxy Metabolites. Stem Cells.

[187] Ryan A.S., Astwood J.D., Gautier S., Kuratko C.N., Nelson E.B., Salem N. (2010). Effects of Long-Chain Polyunsaturated Fatty Acid Supplementation on Neurodevelopment in Childhood: A Review of Human Studies. Prostaglandins Leukot Essent Fat. Acids.

[188] Bertrand P.C., O’Kusky J.R., Innis S.M. (2006). Maternal Dietary (n-3) Fatty Acid Deficiency Alters Neurogenesis in the Embryonic Rat Brain. J. Nutr..

[189] Harvey L., Boksa P. (2014). Additive Effects of Maternal Iron Deficiency and Prenatal Immune Activation on Adult Behaviors in Rat Offspring. Brain Behav. Immun..

[190] Hsieh H.Y., Chen Y.C., Hsu M.H., Yu H.R., Su C.H., Tain Y.L., Huang L.T., Sheen J.M. (2020). Maternal Iron Deficiency Programs Offspring Cognition and Its Relationship with Gastrointestinal Microbiota and Metabolites. Int. J. Environ. Res. Public Health.

[191] Berglund S.K., Torres-Espínola F.J., García-Valdés L., Segura M.T., Martínez-Zaldívar C., Padilla C., Rueda R., Pérez García M., McArdle H.J., Campoy C. (2017). The Impacts of Maternal Iron Deficiency and Being Overweight during Pregnancy on Neurodevelopment of the Offspring. Br. J. Nutr..

[192] Eyles D.W., Feron F., Cui X., Kesby J.P., Harms L.H., Ko P., McGrath J.J., Burne T.H.J. (2009). Developmental Vitamin D Deficiency Causes Abnormal Brain Development. Psychoneuroendocrinology.

[193] Becker A., Eyles D.W., McGrath J.J., Grecksch G. (2005). Transient Prenatal Vitamin D Deficiency Is Associated with Subtle Alterations in Learning and Memory Functions in Adult Rats. Behav. Brain Res..

[194] Harms L.R., Eyles D.W., McGrath J.J., Mackay-Sim A., Burne T.H.J. (2008). Developmental Vitamin D Deficiency Alters Adult Behaviour in 129/SvJ and C57BL/6J Mice. Behav. Brain Res..

[195] Kesby J.P., O’Loan J.C., Alexander S., Deng C., Huang X.F., McGrath J.J., Eyles D.W., Burne T.H.J. (2012). Developmental Vitamin D Deficiency Alters MK-801-Induced Behaviours in Adult Offspring. Psychopharmacology.

[196] Watkins A.J., Rubini E., Hosier E.D., Morgan H.L. (2020). Paternal Programming of Offspring Health. Early Hum. Dev..

[197] Mcmahon D.M., Liu J., Zhang H., Torres M.E., Best R.G. (2013). Maternal Obesity, Folate Intake, and Neural Tube Defects in Offspring. Birth Defects Res. A Clin. Mol. Teratol..

[198] Ryan D.P., Henzel K.S., Pearson B.L., Siwek M.E., Papazoglou A., Guo L., Paesler K., Yu M., Müller R., Xie K. (2018). A Paternal Methyl Donor-Rich Diet Altered Cognitive and Neural Functions in Offspring Mice. Mol. Psychiatry.

[199] Korgan A.C., Foxx C.L., Hashmi H., Sago S.A., Stamper C.E., Heinze J.D., O’Leary E., King J.L., Perrot T.S., Lowry C.A. (2022). Effects of Paternal High-Fat Diet and Maternal Rearing Environment on the Gut Microbiota and Behavior. Sci. Rep..

[200] Trujillo-Villarreal L.A., Cruz-Carrillo G., Angeles-Valdez D., Garza-Villarreal E.A., Camacho-Morales A. (2024). Paternal Prenatal and Lactation Exposure to a High-Calorie Diet Shapes Transgenerational Brain Macro- and Microstructure Defects, Impacting Anxiety-Like Behavior in Male Offspring Rats. eNeuro.

[201] Hollander J., McNivens M., Pautassi R.M., Nizhnikov M.E. (2019). Offspring of Male Rats Exposed to Binge Alcohol Exhibit Heightened Ethanol Intake at Infancy and Alterations in T-Maze Performance. Alcohol.

[202] Koabel J., McNivens M., McKee P., Pautassi R., Bordner K., Nizhnikov M. (2021). The Offspring of Alcohol-Exposed Sires Exhibit Heightened Ethanol Intake and Behavioral Alterations in the Elevated plus Maze. Alcohol.

[203] Meek L.R., Myren K., Sturm J., Burau D. (2007). Acute Paternal Alcohol Use Affects Offspring Development and Adult Behavior. Physiol. Behav..

[204] Ledig M., Misslin R., Vogel E., Holownia A., Copin J.C., Tholey G. (1998). Paternal Alcohol Exposure: Developmental and Behavioral Effects on the Offspring of Rats. Neuropharmacology.

[205] Nieto S.J., Harding M.J., Nielsen D.A., Kosten T.A. (2022). Paternal Alcohol Exposure Has Task- and Sex-Dependent Behavioral Effect in Offspring. Alcohol Clin. Exp. Res..

[206] Rathod R.S., Ferguson C., Seth A., Baratta A.M., Plasil S.L., Homanics G.E. (2020). Effects of Paternal Preconception Vapor Alcohol Exposure Paradigms on Behavioral Responses in Offspring. Brain Sci..

[207] Luo G., Wei R., Wang S., Luo G., Wei R. (2017). Paternal Bisphenol a Diet Changes Prefrontal Cortex Proteome and Provokes Behavioral Dysfunction in Male Offspring. Chemosphere.

[208] Fan Y., Tian C., Liu Q., Zhen X., Zhang H., Zhou L., Li T., Zhang Y., Ding S., He D. (2018). Preconception Paternal Bisphenol A Exposure Induces Sex-Specific Anxiety and Depression Behaviors in Adult Rats. PLoS ONE.

[209] Fan Y., Ding S., Ye X., Manyande A., He D., Zhao N., Yang H., Jin X., Liu J., Tian C. (2013). Does Preconception Paternal Exposure to a Physiologically Relevant Level of Bisphenol A Alter Spatial Memory in an Adult Rat?. Horm. Behav..

